# DNA Methylation-Guided Prediction of Clinical Failure in High-Risk Prostate Cancer

**DOI:** 10.1371/journal.pone.0130651

**Published:** 2015-06-18

**Authors:** Kirill Litovkin, Aleyde Van Eynde, Steven Joniau, Evelyne Lerut, Annouschka Laenen, Thomas Gevaert, Olivier Gevaert, Martin Spahn, Burkhard Kneitz, Pierre Gramme, Thibault Helleputte, Sofie Isebaert, Karin Haustermans, Mathieu Bollen

**Affiliations:** 1 Laboratory of Biosignaling & Therapeutics, KU Leuven Department of Cellular and Molecular Medicine, University of Leuven, Leuven, Belgium; 2 Urology, University Hospitals Leuven & KU Leuven Department of Development and Regeneration, University of Leuven, Leuven, Belgium; 3 Pathology, University Hospitals Leuven & KU Leuven Department of Imaging and Pathology, University of Leuven, Leuven, Belgium; 4 KU Leuven Biostatistics and Statistical Bioinformatics Centre, University of Leuven, Leuven, Belgium; 5 Stanford Center for Cancer Systems Biology, Stanford University School of Medicine, Stanford, California, United States of America; 6 Laboratory of Cancer Data Fusion, KU Leuven Department of Oncology, University of Leuven, Leuven, Belgium; 7 Department of Urology, University Hospital Bern, Inselspital, Bern, Switzerland; 8 Department of Urology and Paediatric Urology, University Hospital Würzburg, Würzburg, Germany; 9 DNAlytics SA, Chemin du Cyclotron 6, 1348 Louvain-la-Neuve, Belgium; 10 Radiation Oncology, University Hospitals Leuven & KU Leuven Department of Oncology, University of Leuven, Leuven, Belgium; Innsbruck Medical University, AUSTRIA

## Abstract

**Background:**

Prostate cancer (PCa) is a very heterogeneous disease with respect to clinical outcome. This study explored differential DNA methylation in a priori selected genes to diagnose PCa and predict clinical failure (CF) in high-risk patients.

**Methods:**

A quantitative multiplex, methylation-specific PCR assay was developed to assess promoter methylation of the *APC*, *CCND2*, *GSTP1*, *PTGS2* and *RARB* genes in formalin-fixed, paraffin-embedded tissue samples from 42 patients with benign prostatic hyperplasia and radical prostatectomy specimens of patients with high-risk PCa, encompassing training and validation cohorts of 147 and 71 patients, respectively. Log-rank tests, univariate and multivariate Cox models were used to investigate the prognostic value of the DNA methylation.

**Results:**

Hypermethylation of *APC*, *CCND2*, *GSTP1*, *PTGS2* and *RARB* was highly cancer-specific. However, only *GSTP1* methylation was significantly associated with CF in both independent high-risk PCa cohorts. Importantly, trichotomization into low, moderate and high *GSTP1* methylation level subgroups was highly predictive for CF. Patients with either a low or high *GSTP1* methylation level, as compared to the moderate methylation groups, were at a higher risk for CF in both the training (Hazard ratio [HR], 3.65; 95% CI, 1.65 to 8.07) and validation sets (HR, 4.27; 95% CI, 1.03 to 17.72) as well as in the combined cohort (HR, 2.74; 95% CI, 1.42 to 5.27) in multivariate analysis.

**Conclusions:**

Classification of primary high-risk tumors into three subtypes based on DNA methylation can be combined with clinico-pathological parameters for a more informative risk-stratification of these PCa patients.

## Introduction

Over the past two decades, the widespread implementation of serum prostate-specific antigen (PSA) testing has led to a dramatic increase in the diagnosis of prostate cancer (PCa) [1]. However, many of the PSA-diagnosed tumors are clinically irrelevant. Only about a quarter of the patients with newly diagnosed PCa are considered to be at high risk of developing fatal disease, manifested by Clinical Failure (CF) and cancer-related death (CRD) [2–4]. According to the European Association of Urology (EAU) and the National Comprehensive Cancer Network (NCCN) guidelines, these high-risk PCa patients are defined by clinical stage ≥T3a, a biopsy Gleason score of 8–10 and/or a serum PSA level >20 ng/ml [5,6]. Nevertheless, 62–84% of the high-risk PCa patients experience cancer-specific survival of at least 15 years after radical prostatectomy (RP), demonstrating that not all patients in this group have a poor prognosis [7]. This heterogeneous clinical outcome within the high-risk group is potentially explained by the use of risk stratification models that do not take into account underlying (epi)genetic and molecular characteristics of the tumor which determine the presence of micrometastases. Therefore, one of the main challenges in contemporary PCa research is to identify biomarkers that improve the prediction of CF and CRD. A better characterization of patients with high-risk PCa at the molecular level should allow a more personalized medicine, matching treatment intensity to disease aggressiveness and expected prognosis. However, to date there is no established clinical indication for using molecular prediction tools.

It is now well recognized that both mutations and epigenetic alterations, in particular differential DNA methylation, play a role in carcinogenesis [8]. DNA methylation, which occurs mainly on cytosine residues in a sequence context of CpG dinucleotides, takes place at different regions in the genome, i.e. at promoter CpG islands (promoter-associated CpG-dense regions), promoter CpG island shores (region with lower CpG density in close proximity of CpG island), gene bodies and repetitive sequences [9]. In the adult human genome most CpGs are methylated, with the exception of the promoter CpG islands and shores. It is generally accepted that PCa is associated with alteration of these patterns, encompassing genome-wide hypomethylation as well as promoter-specific hypermethylation [8–12]. A global hypomethylation is detected at many genomic loci, including repetitive elements and gene bodies, contributing to genome instability and spurious transcriptional initiations, respectively. Promoter-associated hypermethylation is associated with gene silencing and promotes PCa progression by the silencing of tumor-suppressor genes [8,13]. In PCa, various hypermethylated genes have been identified, with *GSTP1* being the most frequently altered and studied [13].

With the present study, we aimed to develop a reliable quantitative assay to simultaneously determine the promoter methylation levels of the a priori selected PCa-linked genes *APC*, *CCND2*, *GSTP1*, *RARB* and *PTGS2*, and to evaluate their diagnostic and prognostic value for high-risk PCa patients [13].

## Materials and Methods

### Patients and sample collection

Patients with high-risk PCa were selected according to the criteria adopted by the EAU and NCCN, i.e. a clinical stage ≥T3a, a biopsy Gleason score of 8–10 and/or PSA levels >20 ng/ml [5,6]. Formalin-fixed, paraffin-embedded (FFPE) normal prostate and PCa tissues were obtained from the University Hospitals Leuven (UHL, Leuven, Belgium) and the University Hospital of Würzburg (UHW, Würzburg, Germany). At the UHL samples were obtained from patients with benign prostatic hyperplasia (BPH, n = 42) or high-risk PCa (PCa2, n = 71). Samples from high-risk PCa patients were also obtained from UHW (PCa1, n = 147). Preoperative staging in both cohorts included a digital rectal examination, an abdominopelvic-computed tomography (CT) scan and a bone scan. Neoadjuvant hormonal, radiation or chemotherapy treatment were an exclusion criterion. Staging and grading of prostate cancer samples (whole mount sections, 4 mm intervals) were performed according to the 2002 TNM classification and the Gleason grading system, as previously described [14]. Follow-up was performed every 3 months for the first 2 years after surgery, every 6 months in the following 3 years, and annually thereafter. CF was declared when either local recurrence or distant metastases were histologically proven or confirmed by CT or bone scan. The clinico-pathological characteristics of all cohorts are described in Table 1. Of the patients of PCa1 and PCa2 cohorts, 84% and 21%, respectively, received adjuvant treatments (radiotherapy and/or hormonal therapy). The study was approved by the Medical Ethics Commission of University Hospital Leuven. The latter granted permission to perform this retrospective study without informed consent because only archived PCa samples (left-over FFPE blocks) were used. All samples were analyzed anonymously.

**Table 1:** Clinico-pathological characteristics of the cohorts.

Clinical variable	PCa1 cohort	PCa2 cohort
Country of origin	Germany (UHW)	Belgium (UHL)
Number of patients	147	71
Median age* (range), y	65 (43–81)	66 (46–76)
Median preoperative PSA, ng/ml, (range)	38.30 (3.00–597.00)	19.90 (2.70–141.00)
Surgical margins, n (%)		
Positive	91 (62)	28 (39)
N.A	8 (5)	0 (0)
Pathological T stage, n (%)		
pT2	13 (9)	19 (27)
pT3a	35 (24)	29 (41)
pT3b	52 (35)	19 (27)
pT4	38 (26)	3 (4)
N.A.	9 (6)	1 (1)
Lymph nodes, n (%)		
Positive	56 (41)	8 (11)
N.A.	10 (7)	0 (0)
Gleason score, n (%)		
2–6	56 (38)	21 (30)
7	42 (28)	33 (46)
8–10	48 (33)	17 (24)
N.A.	1 (1)	0 (0)
Number of CF (%)	30 (20)	13 (18)
Median follow-up (range), y	6.85 (0.08–12.83)	11.50 (1.42–18.83)

*Median age of the patients with benign prostatic hyperplasia (n = 42) were 71 years (range, 48 to 94 years); CF, clinical failure; N.A., not available; PCa1 and 2, cohorts 1 and 2 of patients with high-risk prostate cancer; PSA, prostate-specific antigen; y, year; UHL, University Hospital Leuven and UHW, University Hospital Würzburg.

### Cell culture

Human prostate PC-3 (CRL-1435, American Type Culture Collection (ATCC), Rockville, MD, USA), LNCaP (ATCC, CRL-1740) and DU 145 (ATCC, HTB-81) cells were cultured as monolayers in 50% Dulbecco’s modified Eagle’s medium (DMEM) and 50% Ham’s F12, RPMI1640 and DMEM, respectively, supplemented with 10% fetal calf serum. PZ-HPV-7 cells (ATCC, CRL-2221), an immortalized cell line derived from normal human prostate cells, were cultured in keratinocyte-serum free medium supplemented with 5 ng/ml human recombinant epidermal growth factor and 0.05 mg/ml bovine pituitary extract. The benign prostatic Hyperplasia BPH-1 cells were kindly provided by Prof. J. Swinnen (KU Leuven, Belgium) and were maintained in RPMI medium 1640 plus 10% fetal calf serum [15].

### DNA extraction and bisulfite conversion

Whole blood human genomic DNA was purchased from Clontech Laboratories, Inc, Mountain View, CA, USA. From cell lines genomic DNA was extracted using the GenElute Mammalian Genomic DNA Purification Kit (Sigma-Aldrich, St. Louis, MO, USA). In the BPH cohort, genomic DNA was extracted from the whole paraffin section, using the WaxFreeTM DNA kit (TrimGen, Sparks, MD, USA). For both PCa cohorts, the FFPE block with the largest tumor area was retrieved, and areas with >90% cancerous tissue, comprising both tumor epithelial and tumor-associated stromal cells, were marked by the same uro-pathologist and subsequently macrodissected. Isolation of genomic DNA was performed following a standard phenol-chlorophorm procedure. Next, genomic DNA (~500 ng) from each sample was bisulfite-converted using the EZ DNA methylation kit (Zymo Research Corp., Orange, CA, USA) according to the manufacturer's protocol, and eluted in 25 μl H_2_O.

### Methylation-independent (MI) PCR and cloning

MI primers containing maximally one CpG site close to the 5’ end were designed to amplify 100–200 base-pair (bp) fragments around the transcription start site of *APC*, *CCND2*, *GSTP1*, *PTGS2* and *RARB* (Table A in S1 File, Figure A in S1 File). MI-PCR was performed as previously described [16]. Subsequently, amplified fragments were cloned in DH5α competent cells (Invitrogen Ltd, Paisley, UK), using the pGEM-T Easy Vector System (Promega Corporation, Madison, WI, USA) and about 4 colonies were randomly chosen and analyzed by dideoxynucleotide sequencing. Plasmids with the DNA inserts corresponding to fully methylated (derived from LNCaP or PC-3 cells) or unmethylated (derived from human whole blood) promoter regions after bisulfite conversion, denoted as plasmids pM and pU, respectively, were selected for further use in the second step of the quantitative multiplex methylation-specific PCR (QM-MSP) assay [16].

### QM-MSP assay

A QM-MSP assay was developed to quantify the promoter methylation state of *APC*, *CCND2*, *GSTP1*, *PTGS2* and *RARB* (Figure A in S1 File). A detailed protocol is described in [15], and all primer sets are defined in Table A in S1 File. Briefly, in the first PCR reaction a mixture of validated gene-specific primers was used to co-amplify promoters of the *GSTP1*, *CCND2*, *RARB*, *PTGS2* and *APC* genes independent of their methylation status (MI primer sets in Table A in S1 File). To ensure that the QM-MSP can be used with highly fragmented DNA, which is often problematic in DNA isolated from FFPE tissue, the multiplex primers were designed to amplify PCR fragments of ≈ 100–200 bp. In the second PCR reaction, the absolute quantification of methylated and unmethylated DNA fragments was separately performed for each gene with the validated quantitative methylated and unmethylated specific primers (MSP and USP primers sets, respectively, in Table A in S1 File, Figure B in S1 File). Finally, to calculate the % of DNA methylation the amount of total DNA was derived from the sum of methylated and unmethylated DNA (U+M). Only samples that contain > 3000 gene copies after pre-amplification were accepted for quantification as described in [16]. The methylation of *GSTP1*, *APC* and *CCND2* was quantified in 147 and 70 samples from the PCa1 and PCa2 cohorts, respectively. *PTGS2* was quantified in 147 and 66 samples and *RARB* in 146 and 66 samples from the PCa1 and PCa2 cohorts, respectively.

### Immunohistochemistry

FFPE sections of cohort PCa2 were stained on a BOND MAX autostainer (Leica). Briefly, paraffin-embedded sections were first dewaxed, and antigen retrieval was performed in BOND epitope-retrieval solution 1 (Leica). Mouse monoclonal 3F2 anti-GSTP1 (1:2000, 3369) and rabbit monoclonal anti-ERG (1:100, ab92513) were purchased from Cell Signaling Technology (Danvers, MA) and Abcam (Cambridge, UK), respectively. Slides were analyzed by light microscopy, reviewed and scored by an uropathologist, according the Allred method [17]. The Allred score is a semi-quantitative system that takes into account the proportion of positive cells (scale of 0–5) and staining intensity (scale of 0–3). The proportion and intensity score were summed to obtain the total scores of 0, 2–8. A score of 0–2 was considered as negative, whereas 3–8 was taken as positive [17].

### Statistical analysis

The BPH cohort was used to determine baseline methylation levels for all DNA methylation markers. The receiver-operating-characteristics (ROC) analysis, sensitivity, specificity, and positive/negative predictive value were determined using MedCalc for Windows, version 12.5 (MedCalc Software, Ostend, Belgium). The association between methylation (as continuous variable) and clinico-pathological parameters (Gleason score and pathological stage), ERG and GSTP1 immunostainings and stroma content was explored using the Mann-Whitney U-test (for two categories) or Kruskal-Wallis test (for more than two categories). Fisher exact test is used for the association between two categorical variables. Correlations between methylation of different genes were estimated by Pearson’s correlation coefficient (*r*).

Categorization of DNA methylation for risk-classification was based on Cox models. The functional relationship between the extent of DNA methylation and time-to-event outcomes (CF) was explored by comparing a linear trend to quadratic and cubic-splines based functions using the likelihood ratio test [18]. One or two cut-off values were determined in case of non-linearity. The first cut-off value was determined by considering all possible dichotomizations, whereas the second one was determined by fixing the first cut-off and considering all possible trichotomizations. Both model fit (likelihood) and clinical outcome per data set were considered in the final selection of these cut-off values.

The difference in risk between methylation groups was analyzed by univariate Cox models and the log-rank test. Results are presented by means of hazard ratio (HR) and their 95% confidence intervals (CI), and with a graphical representation provided by plotting the Kaplan-Meier estimates. Multivariate Cox analyses were used to design clinico-pathological models with and without the methylation markers. The predictive accuracy of both models was evaluated by means of the Concordance Probability Estimate (CPE), an AUC-like index for time-to-event data, with values between 0.5 (no predictive value) and 1 (perfect predictive value) [19]. To ensure that the measured CPE is reproducible on out-of-sample patients, repeated random sub-sampling cross-validation was used. The categorization of DNA methylation described above and the weights of a Cox model were tuned on the training sets and evaluated on the corresponding test sets. We performed 200 random splits of the cohort into 80% training set and 20% test set. Analyses were performed using SAS software, version 9.2 for Windows (SAS Institute Inc., Cary, NC, USA). *P*-values <0.05 were considered statistically significant.

## Results

### Methylation of the five marker genes in human prostate cell lines and whole blood

Gene-specific primer pairs were designed to amplify the CpG islands in the promoter region of *GSTP1*, *APC*, *RARB*, *CCND2*, and *PTGS2*, independent of their methylation status (Table A in S1 File). Amplification of these loci was performed in sodium-bisulphite converted genomic DNA isolated from 5 human prostate cell lines, including three PCa (LNCaP, PC-3 and DU 145) and two benign (PZ-HPV-7 and BPH-1) cell lines, and in genomic DNA isolated from human whole blood from a cancer-free person. DNA bisulfite sequencing of these cloned fragments showed that nearly all CpG dinucleotides were methylated in LNCaP and/or PC-3 cancer cell lines, but not in benign cell lines and blood (illustrated in Figure A in S1 File for *APC*). Next, QM-MSP for the five selected genes was developed as described in the materials and methods section and performed in these six genotypes (Table 2). All genes were completely methylated (≥99% methylation) in the hormone-sensitive LNCaP cells, in accordance with our bisulphite sequencing data and previous studies [20]. In the hormone-refractory cell lines PC-3 and DU 145, the DNA methylation levels were less prominent, as only two genes (*APC* and *PTGS2*) were 100% methylated in PC-3 line, while none of the genes was completely methylated in the DU 145 cells. DNA methylation in the non-malignant genotypes, including BPH-1, PZ-HPV7 and whole blood, was not detected at the *GSTP1*, *RARB*, *PTGS2*, *CCND2*, and *APC* gene loci, except for *CCND2* in BPH-1, which was 13% methylated.

**Table 2:** DNA methylation of the five marker genes in human prostate cell lines and whole blood.

	Human prostate cell lines					
	Cancer			Benign		
Genes	LNCaP	PC-3	DU 145	BPH-1	PZ-HPV	HWB*
*GSTP1*	100	32	7	0	0	0
*APC*	100	100	0	0	0	0
*RARB*	100	0	0	0	0	0
*PTGS2*	100	100	61	0	0	0
*CCND2*	99	30	90	13	0	0

*HWB, genomic DNA was isolated from human whole blood from a cancer-free person.

### Cancer-specific DNA methylation in high-risk PCa

**Fig 1:**
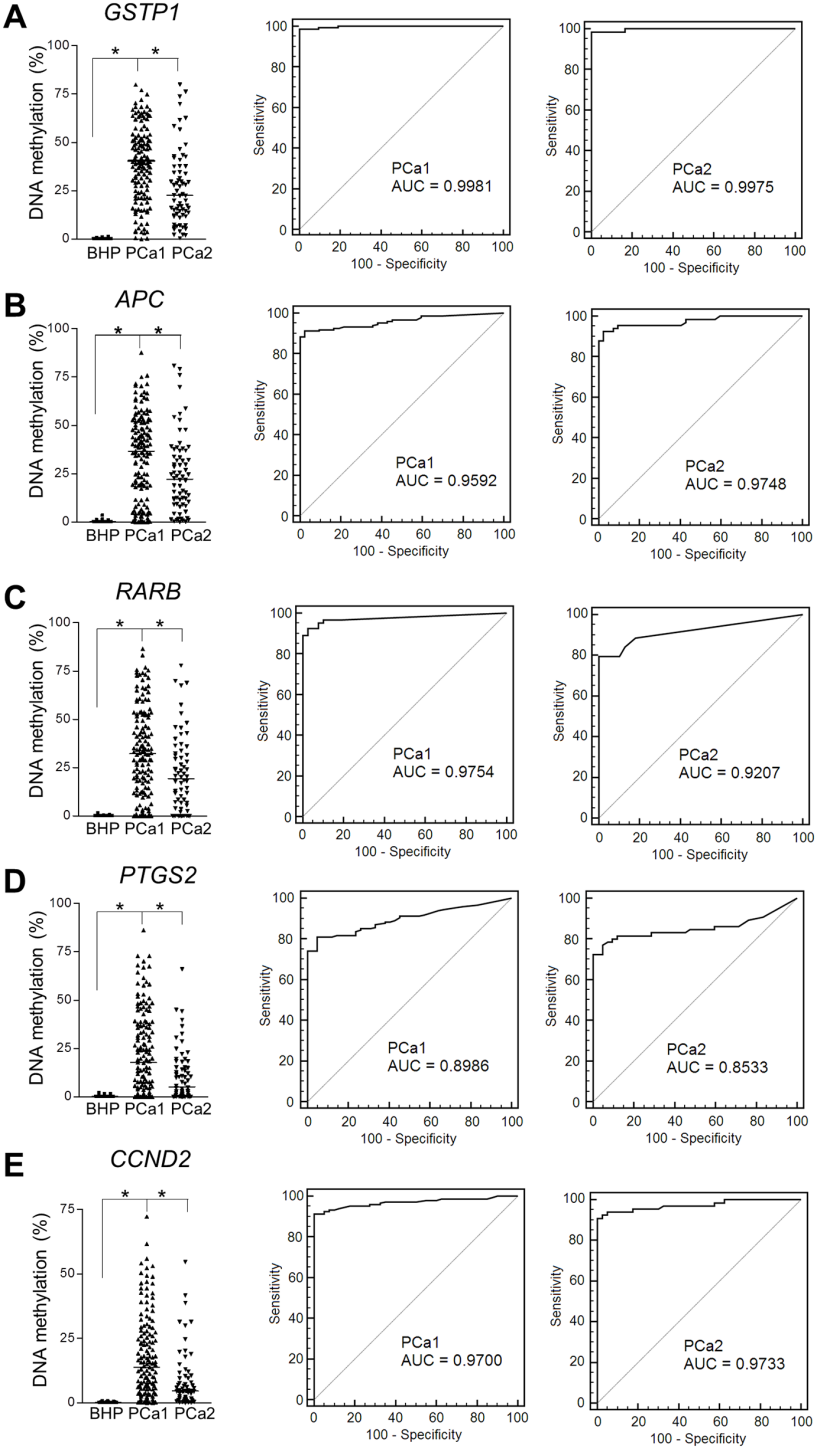
Comparison of DNA-marker methylation in benign prostatic hyperplasia (BPH) and high-risk PCa. The methylation of *RARB*, *GSTP1*, *APC*, *CCND2* and *PTGS2* was determined by QM-MSP in radical prostatectomy samples from 42 patients with BPH and from 147 (PCa1) and 71 (PCa2) patients with high-risk PCa. The data are shown in dot plots for the indicated genes (A-E, left graphs). Methylation levels of every gene in each PCa cohort were significantly higher than in the BPH group, as determined by the Mann-Whitney U-test (*, P<0.001). Receiver-operating-characteristic (ROC) analysis the methylation markers to discern BHP from high-risk prostate cancer samples (A-E; middle, PCa1 and right, PCa2), using the data displayed in the dot plots was performed. Grey line, median; AUC, area under the curve.

Next, the promoter methylation of *APC*, *CCND2*, *GSTP1*, *PTGS2* and *RARB* was quantified in FFPE prostate tissue samples, using the QM-MSP procedure. Samples were derived from the transurethral resection or adenomectomy specimens of 42 patients with BPH and the radical prostatectomy specimens of 218 patients with high-risk PCa. The PCa patients comprised two groups, further denoted as the PCa1 or training cohort (*n* = 147) and PCa2 or validation (*n* = 71) cohort. In the BPH cohort, the average methylation level of these marker genes did not exceed 2% (Fig 1, Table B in S1 File). However, a much higher degree of CpG methylation was detected for all genes in both high-risk PCa cohorts, indicating a cancer-specific methylation of the selected markers (Fig 1, Table C and D in S1 File). With a methylation cutoff value of 1 or 2%, *GSTP1* showed the highest sensitivity in PCa1 (0.99) and PCa2 (0.97) cohorts, at a specificity of 1.00 for the five single markers (Table E in S1 File). Other combinations of marker genes did not further improve the sensitivity without decreasing the specificity (Table E in S1 File). To further assess the accuracy in discriminating high-risk PCa from BPH, receiver-operating-characteristics (ROC) analysis for each of the single markers was performed and the area under the curve (AUC) was calculated (Fig 1). The AUCs ranged from 0.85 (*PTGS2*) to 0.99 (*GSTP1*), confirming cancer-specific methylation, with *GSTP1* methylation as the best classifier.

### Methylation heterogeneity in high-risk PCa

The methylation level of all single genes varied from 0% to ~80%, suggesting a huge inter and intratumor molecular heterogeneity in high-risk prostate tumors (Fig 1). Since the DNA was extracted from areas with >90% cancerous tissue, comprising both tumor-epithelial and tumor-associated stromal cells, we have semi-quantified the stroma content of the tissues from the PCa2 cohort and analysed the correlation with *GSTP1* DNA methylation. The stroma content varied from 10% to 35% with two outliers of 40 and 60% (Table F in S1 File). Importantly, no correlation was found between *GSTP1* DNA methylation and stroma content, as analysed by Spearman (*P*-value, 0.1550), Kruskal-Wallis and Fisher exact tests (Table G in S1 File), indicating that the difference in stroma content is not the underlying cause for inter and intratumor heterogeneity of the DNA methylation. Therefore, our data are consistent with the notion that there is a large inter and intratumor heterogeneity in high-risk PCa at the DNA methylation level.

**Fig 2:**
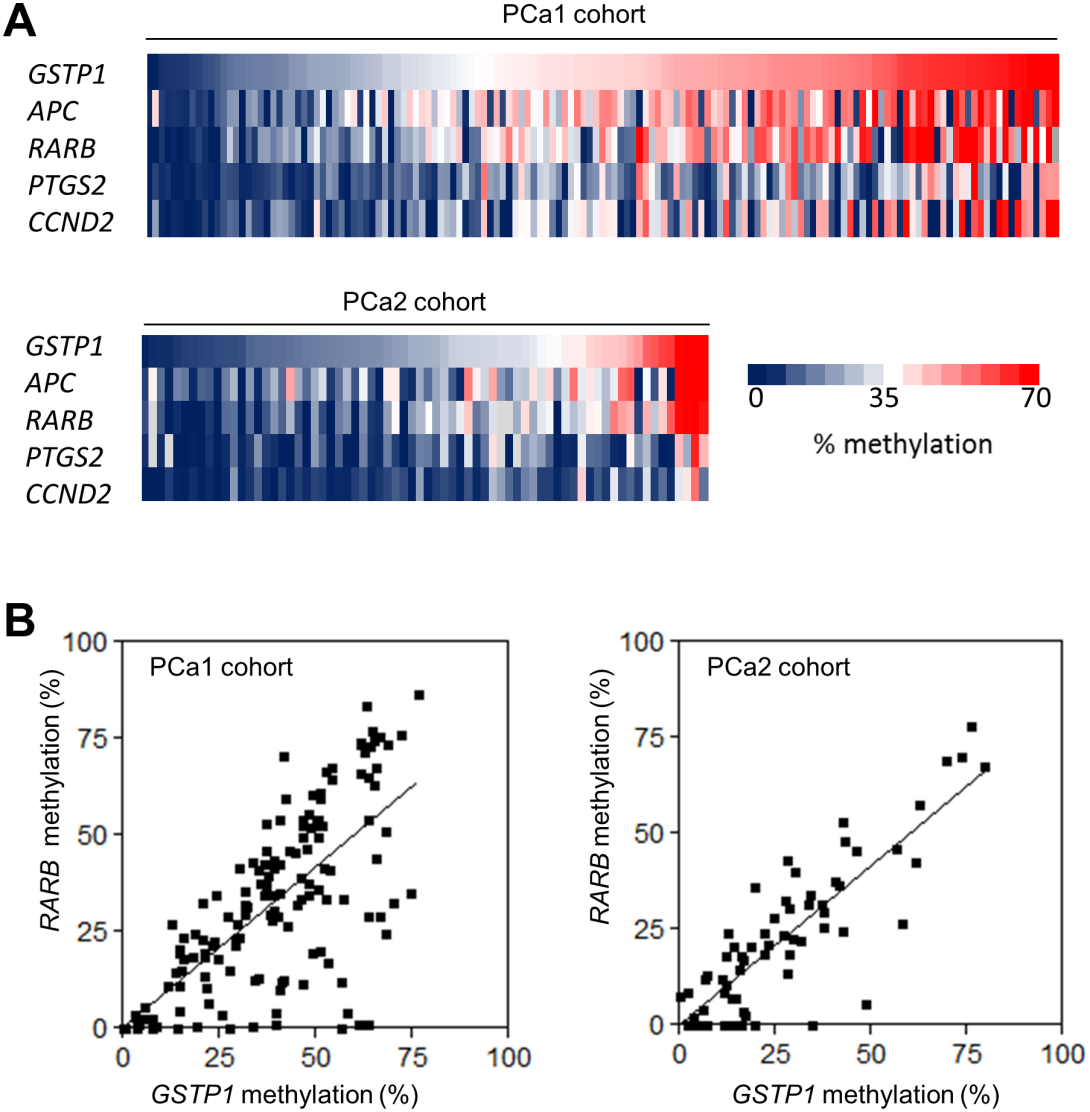
Range of DNA marker methylation in patients with high-risk PCa. Color-scaled representation of DNA methylation in tumors from the PCa1 and PCa2 cohorts. (A) The patients were ordered according to their *GSTP1* methylation value. The % of methylation of the other 4 marker genes is also shown. Grey boxes indicate missing values. (B) Scatter plots of the correlation between *GSTP1* methylation and *RARB* methylation in PCa1 (left panel) and PCa2 (right panel) cohorts.

Interestingly, by ranking the patients of the training and validation cohorts according to the methylation level of *GSTP1*, the most frequently methylated marker gene, we found that the methylation level of the other 4 markers generally followed the same pattern in both cohorts (Fig 2A). In addition, scatter plot analyses between methylation of *GSTP1* and the other genes (Fig 2B and Figure C in S1 File) revealed that tumors that were not methylated at *APC*, *CCND2*, *PTGS2* or *RARB*, were often methylated at *GSTP1*, with levels ranging from ~5 to 70%. However, if the tumors were hypermethylated at *APC*, *CCND2*, *PTGS2* or *RARB*, their methylation degree was generally proportional to the *GSTP1* methylation levels. These findings were corroborated by the highly significant positive Pearson correlation coefficients of the methylation levels of these markers (*r* = 0.45–0.82; *P* < 0.001; Table H in S1 File). Taken together, these data show that the methylation of the five genes is moderately to strongly correlated and most likely occurs in the same cells.

### Promoter hypermethylation versus clinico-pathological parameters

Correlations between the methylation of the gene loci, the pathological stage (pT), and the Gleason score (GS) were evaluated in both PCa cohorts. None of the markers showed a significant association with pT, which was categorized into four groups (pT2, pT3a, pT3b and pT4). For GS, the data were separated into groups of low (GS2-6), intermediate (GS7) and high grade (GS8-10). The median methylation levels for these groups are given in Table 3. Based upon the Kruskal-Wallis and Mann-Whitney U tests, none of the markers were significantly associated with the GS in both cohorts (Table 3 and Tabel I in S1 File). Pairwise comparisons of the GS groups revealed that only *PTGS2* methylation was significantly increased in GS8-10 and GS7, as compared with GS2-6, in PCa1 (Table I in S1 File). *APC* and *RARB* methylations were only significantly higher in GS7, as compared with GS2-6, in PCa2 (Table I in S1 File).

**Table 3:** Correlation analysis of DNA methylation and Gleason score.

		*GSTP1*		*APC*		*RARB*		*PTGS2*		*CCND2*	
Cohort	Gleason score	n	M % (Q1–Q3)	n	M % (Q1–Q3)	n	M % (Q1–Q3)	n	M % (Q1–Q3)	n	M % (Q1–Q3)
**PCa1**	2–6	56	41 (20–53)	56	25 (6–49)	56	27 (12–47)	56	6 (0–26)	56	10 (3–24)
	7	42	42 (29–52)	42	41 (12–53)	41	34 (20–49)	42	19 (6–45)	42	12 (6–28)
	8–10	48	39 (30–52)	48	41 (21–52)	47	35 (19–54)	48	27 (7–39)	48	17 (5–29)
	*P* Value*	0.604		0.135		0.165		0.007		0.402	
**PCa2**	2–6	21	20 (12–30)	21	16 (1–27)	20	8 (0–24)	18	2 (1–10)	21	4 (1–7)
	7	32	26 (15–39)	32	25 (12–39)	30	24 (12–37)	32	12 (1–21)	32	6 (3–11)
	8–10	17	15 (7–43)	17	23 (13–34)	16	16 (7–30)	16	7 (1–11)	17	1 (1–5)
	*P* Value*	0.335		0.134		0.082		0.145		0.060	

Pathological stage was tested without showing significant correlations to hypermethylation. n, number of patients; M = Median DNA methylation (%), Q1, percentile 25; Q3, percentile 75;

*, Kruskal-Wallis test.

### *GSTP1* methylation predicts clinical failure in high-risk PCa patients

Next, we have explored the relationship between the extent of marker methylation and clinical failure, using Cox models [21]. Clinical failure was declared when either local recurrence or distant metastases were histologically proven or confirmed by CT or bone scan. No significant linear relationship was found between each of the five genes and CF. Since it is well established that biomarkers, when applied clinically, are often dichotomized, we used cox models for selecting the optimal cutoffs for each of the single markers [22]. Univariate and multivariate cox regression analysis revealed that dichotomization based on *CCND2*, *GSTP1*, *PTGS2*, or *RARB* methylation was only significantly correlated with CF in one cohort, indicating that it is clinically irrelevant (Table J in S1 File). Nevertheless, we noticed that *GSTP1* methylation was significant associated with CF in PCa1 and PCa2 when different cutoffs were used, i.e, 15% and 50%, respectively (Table J in S1 File). Therefore, we investigated the clinical outcome of multiple *GSTP1* methylation subgroups. The high-risk tumors were categorized into three groups, based upon the *GSTP1* methylation level, i.e. low methylation (LM, methylation <15%), moderate methylation (MM, methylation 15–50%) and high methylation (HM, methylation >50%). Patients with either low or high methylation, as compared to the moderate methylation groups, were at a significantly higher risk for CF in the training PCa1 cohort, as shown by univariate Cox regression analysis (Table 4; HR, 2.96; 95% Cl, 1.38–6.36; *P*-value 0.005). This increased risk was validated in the PCa2 cohort (Table 4; HR, 3.34; 95% Cl, 1.03–10.89; *P*-value 0.045) as well as the combined cohort (Table 4; HR, 2.59; 95% Cl, 1.38–4.87; *P*-value 0.003). In addition, univariate Cox regression analysis of preoperative PSA, GS and pT revealed that only GS was a significant predictor for clinical failure in both cohorts (Table 4). The prognostic power of GS in these cohorts is in agreement with several previous studies [14,23,24].

**Table 4:** Univariate and multivariate Cox regression analysis of clinical failure in high-risk prostate cancer.

	Univariate			Multivariate		
Variable	HR	95% CI	*P*-value	HR	95% CI	*P*-value
**Training Cohort (PCa1)**						
*GSTP1* trichotomized: MM vs LM+HM	2.96	1.38–6.36	0.005	3.65	1.65–8.07	0.001
Pathological T stage 2-3a vs 3b - 4	2.28	0.93–5.61	0.072	1.69	0.67–4.26	0.268
Gleason score 2–7 vs 8–10	3.40	1.63–7.09	0.001	4.82	2.18–10.66	< 0.001
Preoperative PSA continuous	1.00	1.00–1.01	0.640	1.00	0.99–1.01	0.716
**Validation Cohort (PCa2)**						
*GSTP1* trichotomized: MM vs LM+HM	3.34	1.03–10.89	0.045	4.27	1.03–17.72	0.046
Pathological T stage 2-3a vs 3b - 4	7.03	2.14–23.09	0.001	7.15	2.08–24.61	0.002
Gleason score 2–7 vs 8–10	7.49	2.28–24.68	< 0.001	7.12	1.83–27.80	0.005
Preoperative PSA continuous	1.01	1.00–1.03	0.107	1.02	1.00–1.04	0.106
**Combined cohort (PCa1 and 2)**						
*GSTP1* trichotomized: MM vs LM+HM d	2.59	1.38–4.87	0.003	2.74	1.42–5.27	0.003
Pathological T stage 2-3a vs 3b-4	3.99	1.93–8.24	< 0.001	3.82	1.75–8.36	< 0.001
Gleason score 2–7 vs 8–10	4.56	2.43–8.55	< 0.001	4.35	2.28–8.28	< 0.001
Preoperative PSA continuous	1.00	1.00–1.01	0.129	1.00	1.00–1.01	0.388

Hazard Ratio (HR) >1 (<1) indicates higher (lower) risk for the second group. *GSTP1*, % *GSTP1* methylation; CF, clinical failure; CI, confidence interval; HM, high methylation; LM, low methylation; MM, moderate methylation; PCa1 and 2, cohorts 1 (n = 147) and 2 (n = 71) of patients with high-risk prostate cancer; PCa1+2, a combined group of patients from PCa1 and PCa2.

**Fig 3:**
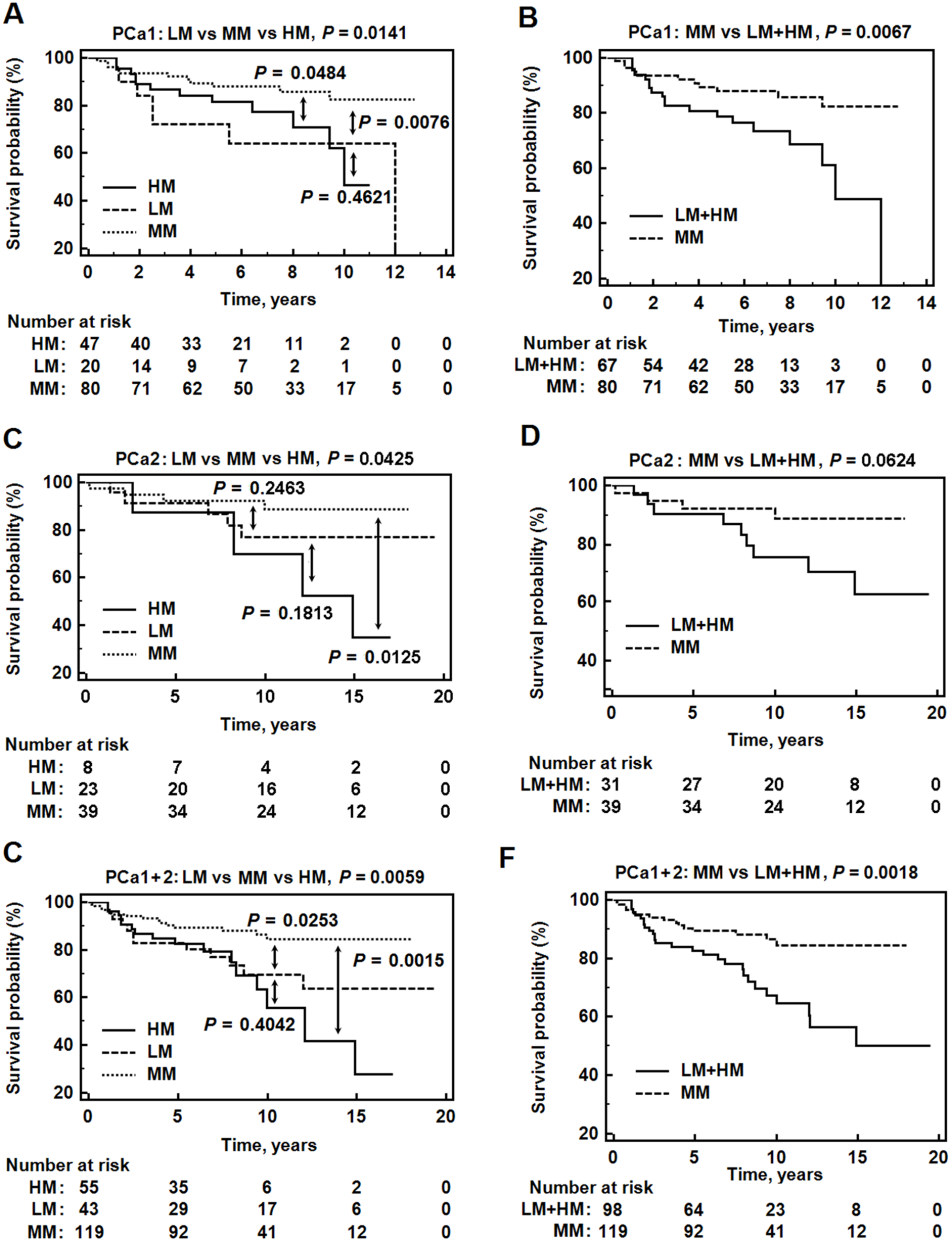
Kaplan-Meier survival plots for patient methylation subgroups. The curves show CF-free survival of patients from the low (LM, <15% *GSTP1* methylation), moderate (MM, 15–50% *GSTP1* methylation) and high (HM, >50% *GSTP1* methylation) groups in PCa1 (A), PCa2 (C) and PCa1+2 (E). Comparison of CF-free survival between the MM and LM+HM groups in PCa1 (B), PCa2 (D) and in PCa1+2 (F) is also shown. CF, clinical failure; *P*, log-rank test *P*-value. *P*-value, log rank test.

To further support the notion that trichotomization of high-risk PCa patients based on their *GSTP1* methylation level improves prediction of clinical outcome, survival probabilities for each group were displayed using Kaplan-Meier plots (Fig 3). This analysis revealed a significant separation in the curves in both the training (log-rank test, *P*-value 0.014) and validation (log-rank test, *P*-value 0.043) cohorts as well as in the combined cohort (log-rank test, *P*-value 0.006) (Fig 3A, 3C and 3E). A comparison of the LM + HM and the MM groups revealed that more than half of the high-risk PCa patients were classified in the MM subgroup and had a much better CF-free survival in PCa1 (log-rank test, *P*-value 0.007) and the combined group (log-rank test, *P*-value 0.002). However, this difference was borderline in the PCa2 cohort (log-rank test, *P*-value 0.062).

Importantly, the trichotomized *GSTP1* methylation emerged as an independent predictor of CF when adjusted for pT, final GS, pre-operative PSA level in both the training (Table 4; HR, 3.65; 95% CI, 1.65–8.07; *P*-value 0.001) and validation cohorts (Table 4, HR, 4.27; 95% CI, 1.03–17.72; *P*-value 0.046), as well as in the joint cohort (Table 4; HR, 2.74; 95% CI, 1.42–5.27; *P*-value 0.003). Among the other tested variables, only the GS also emerged as an independent predictor of CF in both the single and combined cohorts. Thus, DNA methylation has predictive power independent of GS in high-risk PCa.

Next, we compared the predictive value of the model for CF, including all clinico-pathological parameters, with and without trichotomized *GSTP1* methylation, by means of Concordance Probability Estimates (CPEs) [19]. The performance measures were significantly improved by the inclusion of *GSTP1* methylation in the clinical model in both cohorts (Table 5). Thus, the accuracy of the predictive models for CF based on clinico-pathological parameters can be further improved by inclusion of the trichotomized *GSTP1* methylation model.

**Table 5:** Performance of multivariate Cox regression of clinical failure in high risk prostate cancer.

Variable	CPE ± CI*
**Training cohort (PCa1)**	
Three clinical variables	0.684 ± 0.052
Three clinical variables + *GSTP1*	0.717 ± 0.051
*P*-value^$^	0.003
**Validation cohort (PCa2)**	
Three clinical variables	0.747 ± 0.099
Three clinical variables + *GSTP1*	0.797 ± 0.084
*P*-value^$^	0.001

*Condifidence intervals (CI) of the measures on 200 sampled test sets are reported.

^$^ *P*-values were produced by paired t-tests on the 200 measures. CPE, Concordance Probability Estimate; GSTP1, % DNA methylation of *GSTP1* gene; PCa1 and 2, cohorts 1 (n = 147) and 2 (n = 71) of patients with high-risk prostate cancer.

### Methylation-guided risk-stratification of patients with high-risk PCa

**Fig 4:**
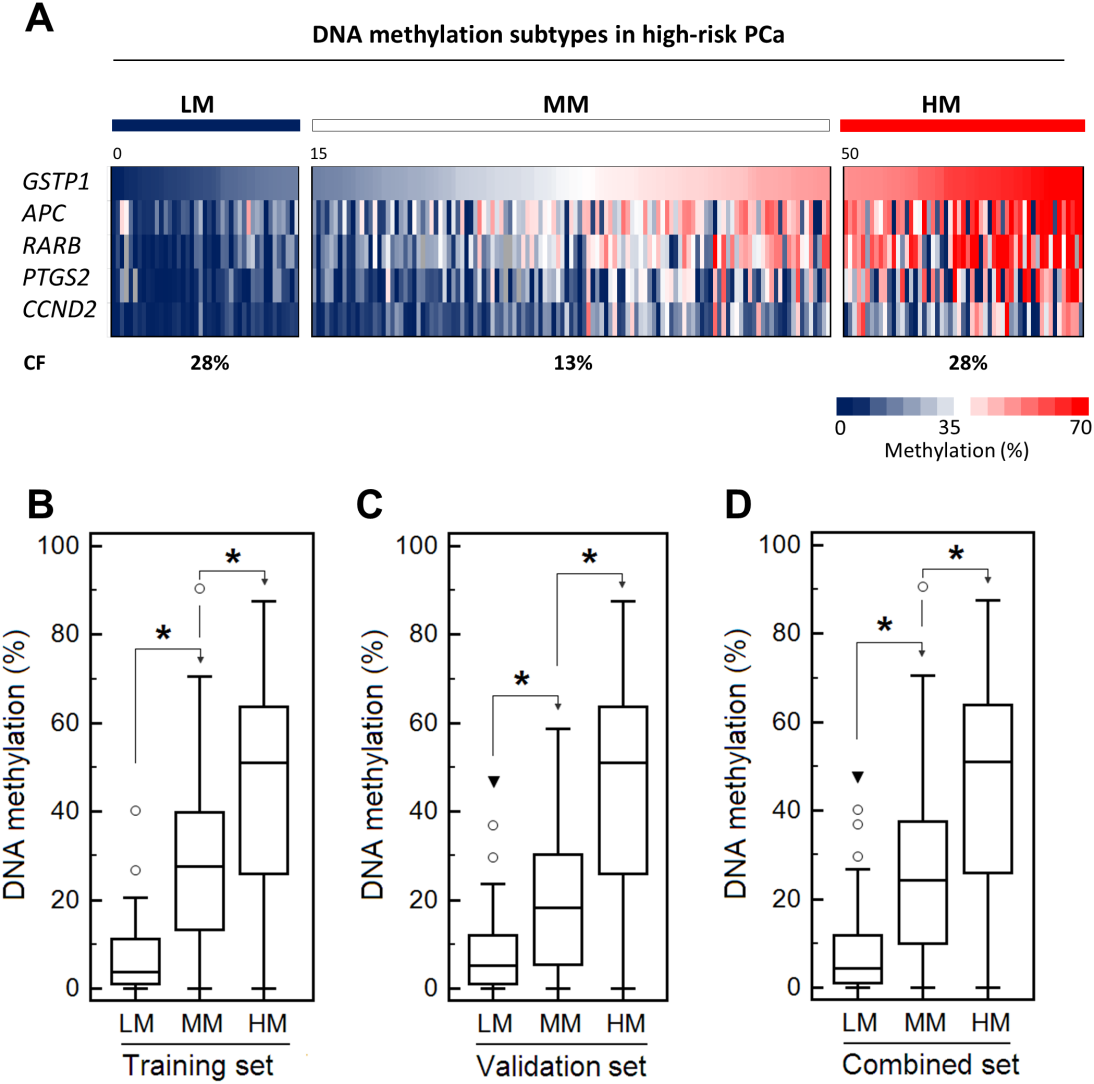
Methylation-guided sub-stratification of patients with high-risk PCa. (A) Color-coded representation of DNA methylation in tumors from the combined PCa1 + PCa2 cohort. The patients were ranked according to their *GSTP1* methylation value and classified into low (LM), moderate (MM) and high (HM) methylation subtypes, based on *GSTP1* methylation levels of < 15%, 15–50% and > 50%. The % of methylation of the other 4 marker genes is also shown. Grey boxes indicate missing values. (B-D) Box-whisker plots of the methylation of the 5 marker genes in the LM, MM and HM groups of the training, validation and combined cohorts. The boxes mark the 25th-75th percentiles, the median value (horizontal line in the boxes), and the minimal and maximal values (whiskers). Circles indicate the “outside” values (defined as those that are larger than the upper quartile plus 1.5 times the interquartile range). A filled triangle indicates a “far out” value (defined as that larger than the upper quartile plus 3 times the interquartile range). (*) *P* < 0.001, Mann-Whitney U test.

Since the survival analyses showed that patients with either low or high levels of *GSTP1* methylation are at a higher risk for CF than those with moderate *GSTP1* methylation levels, we explored the methylation level of the other 4 tested markers in these subgroups. First, we stratified the patients of the training, validation and combined cohorts into three groups, i.e. low (<15%), moderate (15%-50%) and high (>50%) methylation, by ranking them according to the *GSTP1* methylation level (Fig 4A). When the data of all five marker genes were combined, the median methylation values in the LM groups did not exceed 6% and, except for a few outliers, the absolute values were less than 25% in both the single and combined cohorts (Fig 4B–4D). This is consistent with the high Pearson correlation coefficients (Table H in S1 File) and suggests that only a small percentage of the cells in LM tumors were methylated at the marker genes. In contrast, the median methylation values for the marker genes in the MM/HM groups reached 18/45% (training set), 28/50% (validation set) and 25/50% (combined set), respectively. Importantly, when the patients were ranked according to the methylation state of *APC*, *RARB*, *CCND2* or *PTGS2*, their segregation into low, moderate and high methylation subtypes was less manifested, as each of those markers showed a lower sensitivity, as compared with *GSTP1* (Figure D in S1 File). Collectively, these data suggest that only categorization according to the *GSTP1* methylation level defines subgroups with globally low, moderate or high methylation levels in high-risk prostate patients. Patients with either low or high DNA methylation levels are clearly at higher risk for CF than patients with a moderate DNA methylation level (Figure E in S1 File).

### ERG and GSTP1 expression in high-risk PCa

**Fig 5:**
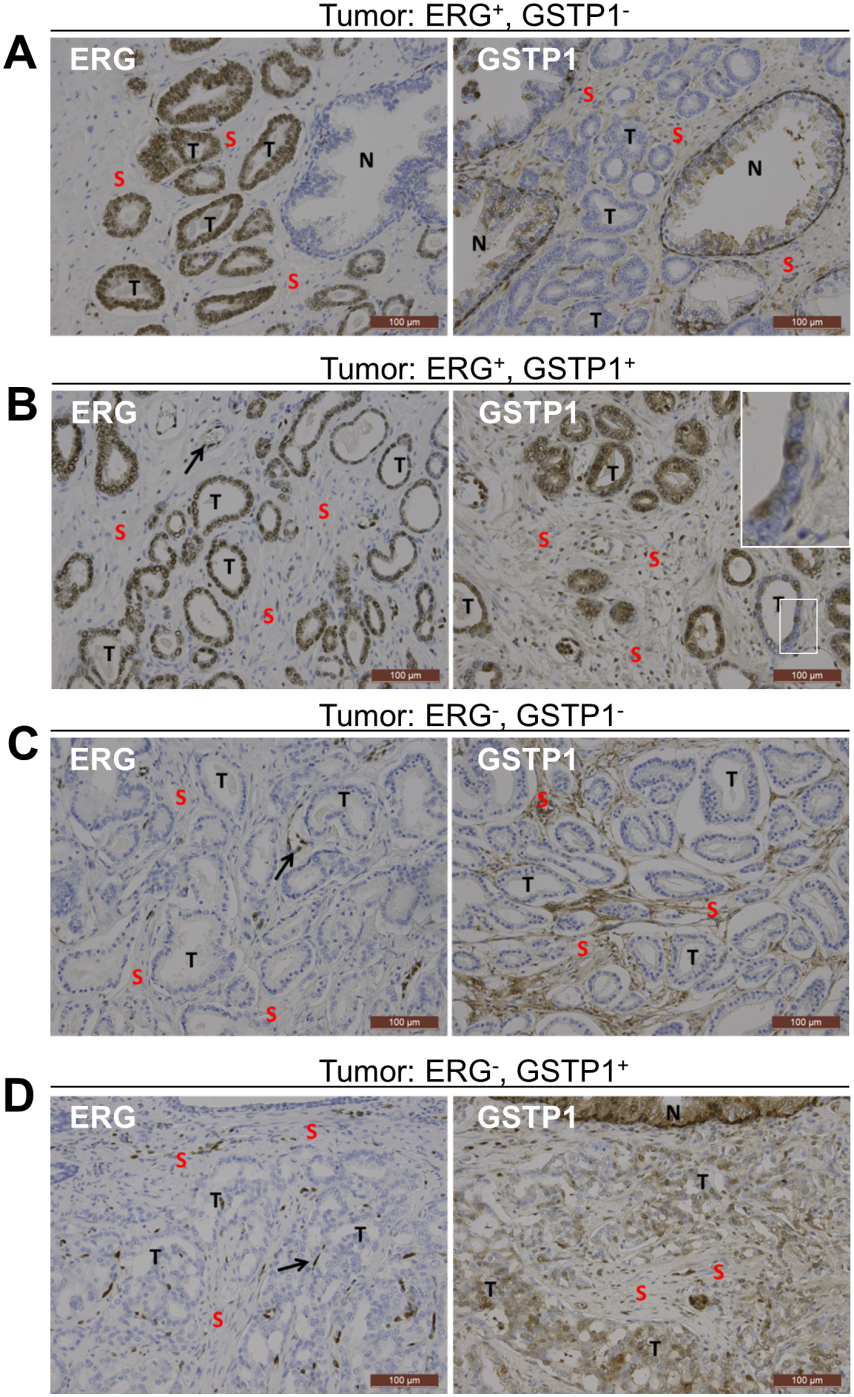
ERG and GSTP1 immunostainings of PCa samples from cohort PCa2. Representative immunohistochemical images of PCa samples are shown that were positive for ERG and negative for GSTP1 (A), positive for both ERG and GSTP1 (B), negative for both ERG and GSTP1(C), and negative for ERG and positive for GSTP1 (D). The internal staining control for ERG is the endothelium (arrows) and for GSTP1 the stromal and/or basal cells of normal prostate glands. N, normal prostate gland; S, Stroma; T, tumor gland. Scale bars equal 100μm.

It is well established that gene fusions, including *TMPRSS2-ERG* fusions with concurrent ERG overexpression, represent the most frequent genetic alterration in PCa [25,26]. Therefore, we have performed ERG stainings in cohort PCa2 and analyzed the association with *GSTP1* methylation. Positive ERG staining was found in 62% of the PCa tissues, which is consistent with prior reported frequencies of ERG fusions (Fig 5, Table F in S1 File) [25,26]. No significant correlation was found between the ERG level and *GSTP1* DNA methylation, as analysed by Kruskal-Wallis and Fisher exact tests (Table G in S1 File).

Next, to explore the correlation between *GSTP1* methylation and expression we performed GSTP1 immunostaining on the samples of cohort PCa2. Positive GSTP1 was only found in 5 tumors, and the other 63 samples were completely negative for GSTP1 in the epithelial tumor cells (Fig 5, Table F in S1 File). Importantly, the basal cells of normal prostate glands and the stromal cells served as internal positive staining controls on each slide. Although hypermethylation of *GSTP1* gene is often associated with a loss of GSTP1 expression, we found no association between GSTP1 staining and GSTP1 DNA methylation (Table G in S1 File). This suggests that the *GSTP1* gene is inactivated by other mechanisms, as reported by others [27,28], or is hypermethylated at a region that was not analyzed in our study. The five tumors that stained positively for GSTP1 showed a mixed population of positive and negative tumor cells, clearly demonstrating intra-tumor heterogeneity at the GSTP1 level (Fig 5). Combining the GSTP1 and ERG stainings, we found that 58% of the tumors stained positive for ERG and negative for GSTP1 (Fig 5A), 4% positive for both ERG and GSTP1 (Fig 5B), 35% negative for both ERG and GSTP1 (Fig 5C), and 3% negative for ERG and positive for GSTP1 (Fig 5D).

## Discussion

### Coordinated cancer-specific methylation in PCa

Comparison of BPH and PCa samples revealed that the methylation of the 5 selected markers is highly PCa-specific, with *GSTP1* being the most frequently methylated marker. Of all analysed PCa specimens, 58% showed methylation at all five genes, and 73% at four loci, encompassing *GSTP1* and at least three other loci. Tumors that were not methylated at *APC*, *CCND2*, *PTGS2* and/or *RARB*, were often methylated at *GSTP1*, with levels ranging from ~5 to 70%. This agrees with *GSTP1* being the most sensitive marker in our study and is consistent with reports indicating that *GSTP1* is one of the most altered and earliest epigenetic event during PCa development [29]. We have also observed that if the tumors were hypermethylated at *APC*, *CCND2*, *PTGS2* or *RARB*, their methylation degree was generally proportional to the *GSTP1* methylation levels, suggesting a common underlying mechanism. Accordingly, Spearman analyses revealed that the methylation of the five marker genes was moderately to strongly positively correlated, in agreement with earlier studies [30,31]. Florl et al. (2004) reported a simultaneous hypermethylation of *GSTP1*, *RARB2*, *RASSF1A* and *APC* [30]. Yegnasubramanian et al. (2004) found a similar correlation for the methylation of *GSTP1*, *APC*, *RASSF1A*, *PTGS2* and *MDR1* [31]. Coordinated methylation has also been found for *RARB* and *TIG1* by Zhang et al. (2004). These authors proposed that the methylation of *TIG1* was a downstream effect of the inactivation of *RARB* [32]. More recently, global methylation profiling has revealed hundreds of differentially methylated DNA regions in PCa, which supports the coordinated hypermethylation of gene sets [33–41]. For example, the 25 most hypermethylated genes in PCa compared to normal tissue, including *GSTP1* and *RARB*, showed sensitivities and specificities between 89 and 100%, respectively, confirming the simultaneous methylation of these markers [37]. Although the molecular mechanism underlying DNA methylation changes in PCa remains unclear, it has been linked to an increased expression of DNA methyltransferases (DNMTs) and the chromatin modifier EZH2, a dysregulation of DNMT–interacting proteins, and a reduced level of the hydroxymethylase TET1 [36,42,43]. Interestingly, *GSTP1*, *APC* and *PTGS2* are known TET1 targets [42,43].

Intriguingly, GSTP1 immunostainings revealed that ~ 90% of the tumors from the PCa2 cohort did not express GSTP1 in any of the epithelial tumor cells. Since the *GSTP1* DNA methylation levels ranged from 0–80%, these data therefore suggest that other mechanisms inactivate the *GSTP1* gene. Accordingly, Jeronimo et al. (2002) found that 62% of microdissected prostate adenocarcinomas with unmethylated *GSTP1* showed no GSTP1 protein expression [27]. Accumulating evidence suggests that the *GSTP1* gene can be downregulated by other mechanisms, involving signaling by the estrogen receptor and the endothelial nitric-oxide synthase complex as well as several miRNAs [28,44,45].

### Inter and intratumor heterogeneity in DNA methylation

Since the methylation level is a measure of the percentage of cells from the dissected area that is differentially methylated and the methylation level of individual genes varied from 0% to ~80%, our data suggest massive inter and intratumor heterogeneity in DNA methylation of high-risk PCa. Importantly, since the stroma content was not correlated with DNA methylation, we can exclude that the difference in stroma content is the underlying cause of this tumor heterogeneity. Also, we have found that the tumors that expressed GSTP1 showed a mixed population of positive and negative cells, hinting at intratumor heterogeneity with respect to GSTP1 expression. Brocks et al. (2014) also reported a high intratumor heterogeneity in DNA methylation in aggressive PCa and concluded that the epigenome variation extends the intratumor heterogeneity of PCa along with genetic variation [46].

About 20% of the high-risk patients from the combined cohort belonged to the LM subtype (Fig 4). In this LM group, the average methylation of all five markers amounted to 7.00 ± 0.54%, which represented a significantly higher value than that of benign samples (average methylation 0.13 ± 0.01%; *P* value < 0.0001 (*t*-test)). This agrees with the high sensitivity of the markers (Fig 1 and Table E in S1 File). We can exclude poor DNA quality as an explanation for the low methylation values, because we amplified an adequate amount of DNA in all LM samples. Since the methylation patterns of the five examined marker genes were strongly correlated, this suggests that the LM tumors only contain a small percentage of cells with hypermethylated genes. These LM patients could only be identified because the methylation in our cohorts was selectively quantified in tumor tissue, which prevents ‘dilution’ of the hypermethylation signal by normal tissue. This suggests that PCa patients with LM tumors will probably not be identified when body fluids, including blood and urine, are used for the detection of marker gene hypermethylation. At present it cannot be excluded that LM tumors were initially hypermethylated, but somehow lost their methylation during the later stages of tumorigenesis. A genome-wide analysis also identified a subset of unmethylated metastatic PCa tumors, which did not co-segregate with clinico-pathological factors [34].

Although the underlying molecular mechanism of the LM subclass remains unclear, we hypothesize that the LM tumors represent a biologically distinct subclass of PCa at the epigenetic and molecular level. We speculate that key enzymes for altering the methylome, including DNMTs and/or TETs, are not affected in these tumors. Since aberrant methylation is thought to contribute to tumorigenesis by repressing transcription of tumor suppressor genes, we speculate that other mechanisms are responsible for the inactivation of the tumor suppressor genes in the LM tumors [20,47]. Finally, we did not find a correlation between the ERG level and *GSTP1* DNA methylation, suggesting hat the LM tumors are not different from the MM or HM tumors with respect to ERG rearrangements. Consistent with this view, Kim et al (2011) found no difference in the methylation status at the *GSTP1* locus in ERG-positive and negative cancers [20]. In contrast, Borno et al. (2012) showed that gene-fusion negative tumors have significantly more methylation alteration events, including hypermethylation of *miR-26a*, as compared to the positive tumors. Kron et al. (2012) found that ERG-positive tumors were more methylated on *CYP26A1*, *TBX15* and *HOXD3* than the ERG-negative tumors [48,49]. Further investigations are needed to unravel the molecular basis of the LM tumors subclass.

### DNA methylation-guided risk-stratification in high-risk PCa

Currently, risk-stratification of PCa patients into low, intermediate and high-risk groups, is exclusively based on clinico-pathological parameters, including PSA, GS and clinical stage. However, these groups, in particular the high-risk group, have a very heterogeneous outcome [7]. In line with these observations, we found a great inter and intratumor heterogeneity with respect to DNA methylation level of the selected marker genes in the high-risk patients. Through explorative statistical analyses we found that the classification of high-risk PCa into LM, MM and HM groups, based on their *GSTP1* methylation level, is significantly associated with the prediction of clinical outcome. Indeed, statistical analysis revealed that LM tumors are not less aggressive than HM tumors, and that the combination of both groups is a strong predictor for CF, independent of established clinico-pathological prognostic factors. In line with these results, the predictive value of the clinico-pathological model for CF significantly increased after addition of *GSTP1* methylation to the model. Our study is the first to show a significant association between *GSTP1* methylation and CF in two independent high-risk cohorts, stressing the clinical relevance of these findings. Since both the LM and HM groups showed a worse prognosis compared to the MM group, this enabled us to further risk-stratify high-risk PCa patients.

Promoter hypermethylation of the *GSTP1* gene is the most common epigenetic alteration in PCa and one of the most extensively studied, in particular for PCa diagnostics [50,51]. *GSTP1* methylation has been detected in over 90% of primary PCa and 70% of prostatic intraepithelial neoplasia (PIN) lesions, but only rarely in normal prostate [50,52]. In addition, *GSTP1* hypermethylation has been detected in urine and serum of PCa patients, and has emerged as the most promising epigenetic diagnostic biomarker for PCa [51]. Although the diagnostic utility of GSTP1 methylation is generally accepted, its prognostic value is unclear. Several groups have evaluated GSTP1 methylation as a prognostic biomarker for PCa, but reported discrepant results. Since it is essential to evaluate a prognostic marker by multivariate analyses to be clinically relevant, we focus here our discussion on prognostic studies with multivariable analyses. Ellinger et al. (2008) and Alumkal et al (2008) did not find an association between *GSTP1* DNA methylation and biochemical recurrence, while Richiardi et al. (2009) and Vasiljevic et al. (2014) found no correlation with mortality [53–56]. In contrast, Rosenbaum et al. (2005) and Devaney et al. (2011) found an inverse correlation between *GSTP1* methylation with disease progression [33,57]. Other studies have reported an association between *GSTP1* methylation and poor patient outcome [58–60]). Bastain et al. (2005) demonstrated that men with clinically localized PCa and detectable hypermethylated *GSTP1* in preoperative serum were at a significantly higher risk for PSA recurrence than those without methylated *GSTP1* [58]. In accordance with these data the group of Sidransky has found an association between *GSTP1* hypermethylation and disease recurrence in early stage PCa [59]. Finally, Clark and co-workers reported that detectable plasma levels of methylated *GSTP1* were associated with a poorer response to chemotherapy in castrate-resistant PCa and a poorer survival [60]. Thus, conflicting data regarding the prognostic value of *GSTP1* have been reported. These are probably due to the differences in the adopted methodologies, sample sizes, (neo)adjuvant treatments and/or outcome measurement criteria [61]. Our data suggest that the discrepancies between these studies may be linked to the non-linear association between *GSTP1* methylation and disease progression. We hypothesize that a categorization into three *GSTP1* methylation level subgroups is required to detect the association with CF. From a clinical point of view, we believe that it is important to determine whether DNA methylation is altered in tumors. Hypermethylated *GSTP1* will generally be associated with a poorer outcome, which could contribute to a rational decision on further patient treatment. If *GSTP1* methylation is not altered, DNA methylation was probably not involved in tumor development and epigenetic drugs are unlikely to be effective.

Our study has multiple strengths. We had an extremely long clinical follow-up of 11 and 7 years in the training and validation sets, respectively. We have obtained consistent results for both patient cohorts, despite their different countries of origin. In addition, the DNA was extracted exclusively from tumor regions, as mapped by the same pathologist, allowing us to study intratumor heterogeneity with respect to DNA methylation. Nevertheless, our study is not devoid of limitations. The groups are not homogeneous regarding the use of adjuvant and salvage radiotherapy or androgen deprivation therapy and, due to the long study period, we cannot exclude changes in staging, surgical techniques and/or secondary treatments. Although the tumor samples were macro-dissected from a well-defined tumor area, they were heterogeneous with respect to stroma content, which may have affected the detected methylation profiles. In addition, CpG sites can be either methylated or unmethylated in each allele, which may have resulted in heterogeneous DNA methylation patterns [62]. Therefore, it should be taken into consideration that MSP-based assays examine the DNA methylation status of the CpG sites that are only present in the primer binding sites and do not necessarily reflect the DNA methylation status of other CpG sites. Hence, the DNA methylation levels in tumor samples depend on the amplicon that is amplified. Despite the fact that our study deals with high-risk cancers, the clinical failure rates were rather limited, which may have obscured the detection of modest effects. Therefore, we cannot exclude that significant methylation effects for *APC*, *CCND2*, *PTGS2* and *RARB* will surface in larger-scale studies. Furthermore, a larger-scale analysis should reveal whether the quadratic relationship between *GSTP1* DNA methylation and CF also applies to the low and intermediate risk groups.

In conclusion, our data suggest that the classification of primary high-risk PCa tumors into DNA methylation subtypes i.e. low, moderate and high DNA methylation level groups, can be combined with clinico-pathological parameters for a more informative risk-stratification.

## Supporting Information

S1 File Contains Figs. A–E and Tables A–J. (DOCX)
