# Supplementary material for: DNA Methylation-Guided Prediction of Clinical Failure in High-Risk Prostate Cancer
Source: PLoS One. 2015 Jun 18;10(6):e0130651. doi: 10.1371/journal.pone.0130651 (PMC4472347; doi:10.1371/journal.pone.0130651)
Supplement: S1 File — (DOCX) [file pone.0130651.s001.docx]

**Supporting information**

**DNA methylation-guided prediction of clinical failure in high-risk prostate cancer**

# Kirill Litovkin^1¶^, Aleyde Van Eynde^1¶*^, Steven Joniau^2^, Evelyne Lerut^3^, Annouschka Laenen^4^, Thomas Gevaert^2^, Olivier Gevaert^5,6^, Martin Spahn^7,8^, Burkhard Kneitz^8^, Pierre Gramme^9^, Thibault Helleputte^9^, Sofie Isebaert^10^, Karin Haustermans^10^, and Mathieu Bollen^1^

^1^Laboratory of Biosignaling & Therapeutics, KU Leuven Department of Cellular and Molecular Medicine, University of Leuven, Leuven, Belgium

^2^Urology, University Hospitals Leuven & KU Leuven Department of Development and Regeneration, University of Leuven, Leuven, Belgium

^3^Pathology, University Hospitals Leuven & KU Leuven Department of Imaging and Pathology, University of Leuven, Leuven, Belgium

^4^KU Leuven Biostatistics and Statistical Bioinformatics Centre, University of Leuven, Leuven, Belgium

^5^Stanford Center for Cancer Systems Biology, Stanford University School of Medicine, Stanford, USA

^6^Laboratory of Cancer Data Fusion, KU Leuven Department of Oncology, University of Leuven, Leuven, Belgium

^7^Department of Urology, University Hospital Bern, Inselspital, Bern, Switzerland

^8^Department of Urology and Paediatric Urology, University Hospital Würzburg, Würzburg, Germany

^9^DNAlytics SA, Chemin du Cyclotron 6, 1348 Louvain-la-Neuve, Belgium

^10^Radiation Oncology, University Hospitals Leuven & KU Leuven Department of Oncology, University of Leuven, Leuven, Belgium

^¶^ These authors contributed equally

**INVENTORY**

This file contains supplementary Figures (5) and Tables (10).

**SUPPLEMENTARY FIGURES**

**Figure A**


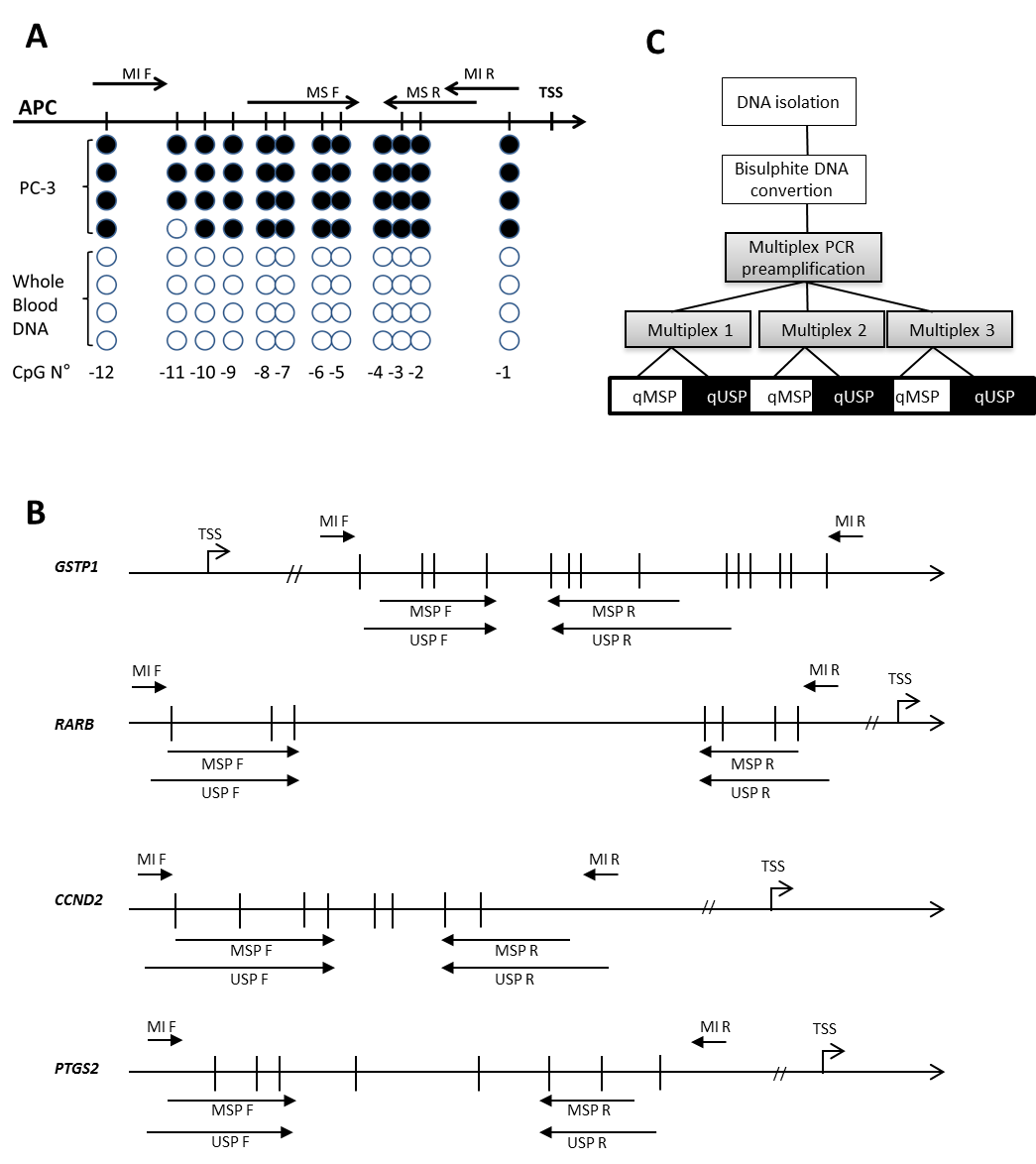


**Supplementary Figure A**. Overview of the two-step quantitative multiplex nested-MSP (QM-MSP). Schematic representation of the localization of the methylation independent (MI_F and MI_R), methylation specific (M_F and M_R) and unmethylation specific (U_F and U_R) PCR primers at the *APC* locus, locating at [Chromosome 5: 112,707,498-112,846,239](http://www.ensembl.org/Homo_sapiens/Location/View?db=core;g=ENSG00000134982;r=5:112707498-112846239) forward strand (CpG -12 at 112,706,668) (A). The other loci are shown in (B). The location of these genes and the first indicated CpG on the scheme is as follow: *GSTP1*, [Chromosome 11: 67,583,595-67,586,660](http://www.ensembl.org/Homo_sapiens/Location/View?db=core;g=ENSG00000084207;r=11:67583595-67586660) forward strand, CpG at 67,583,792; *RARB*, [Chromosome 3: 25,174,332-25,597,932](http://www.ensembl.org/Homo_sapiens/Location/View?db=core;g=ENSG00000077092;r=3:25174332-25597932;t=ENST00000437042) forward strand, CpG at 25,427,980; *CCND*2, [Chromosome 12: 4,273,772-4,305,350](http://www.ensembl.org/Homo_sapiens/Location/View?db=core;g=ENSG00000118971;r=12:4273772-4305350) forward strand, CpG at 4,272,601; *PTGS*2, [Chromosome 1: 186,671,791-186,680,427](http://www.ensembl.org/Homo_sapiens/Location/View?db=core;g=ENSG00000073756;r=1:186671791-186680427) reverse strand, CpG at 186,680,756. CpG is indicated by a vertical line. The drawing is on scale between the first and last indicated CpG. (A) APC PCR products encompassing the studied CpG island were amplified and cloned from LNCaP and human whole blood DNA. The results from bisulphite sequencing of 4 randomly selected clones are shown under the CpG map. White and black lolly-pops represent unmethylated and methylated CpG dinucleotide, respectively*.* (C) A schematic representation of the two-step QM-MSP analysis. TSS, transcriptional start site.

**Figure B**

**
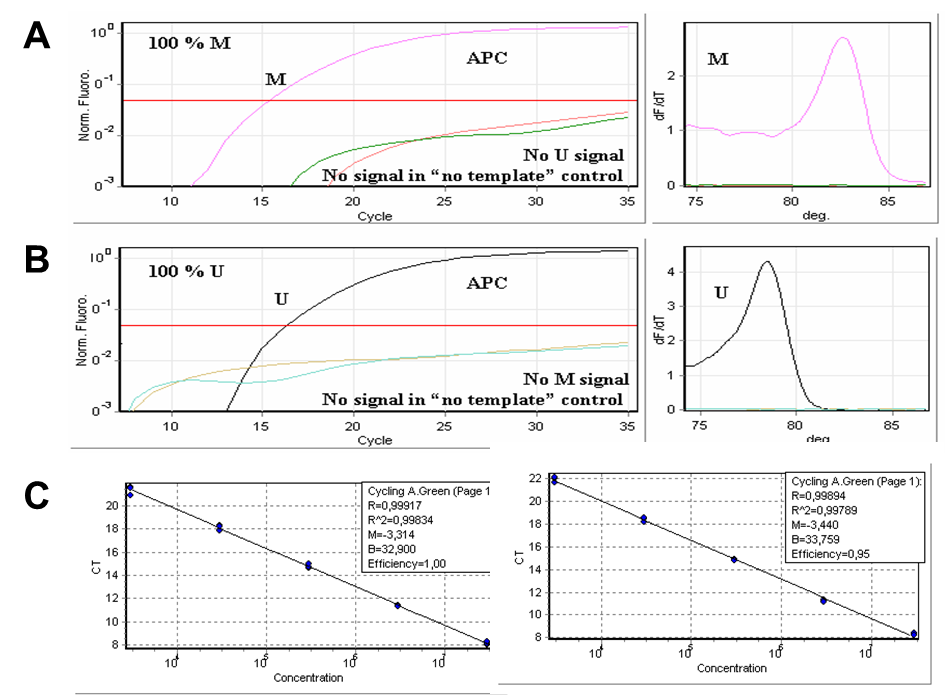
**

**Supplementary Figure B.** Validation of the quantitative Methylation specific (qMSP) and unmethylation specific (qUSP) PCR for the APC gene. (A-B) qMSP (A) and qUSP (B) reactions were performed using both plasmids with the corresponding fully methylated (M) or unmethylated (U) bisulphite-converted DNA as a template for the amplification. PCR cycles plotted against the fluorescence intensity of the PCR product (left panel) and melting curve analysis (right panel) are shown. (C) The correlation coefficient (R^2^) of the standard curves of the qMSP (left panel) and qUSP (right panel) reactions showed linearity (0.99) over the range of the serial dilutions of pM and pU plasmids (3 x 10^3^-10^7^ copies per reaction), respectively. The same approach was used to validate the methylation of the other marker genes (not shown).

**Figure C**

**
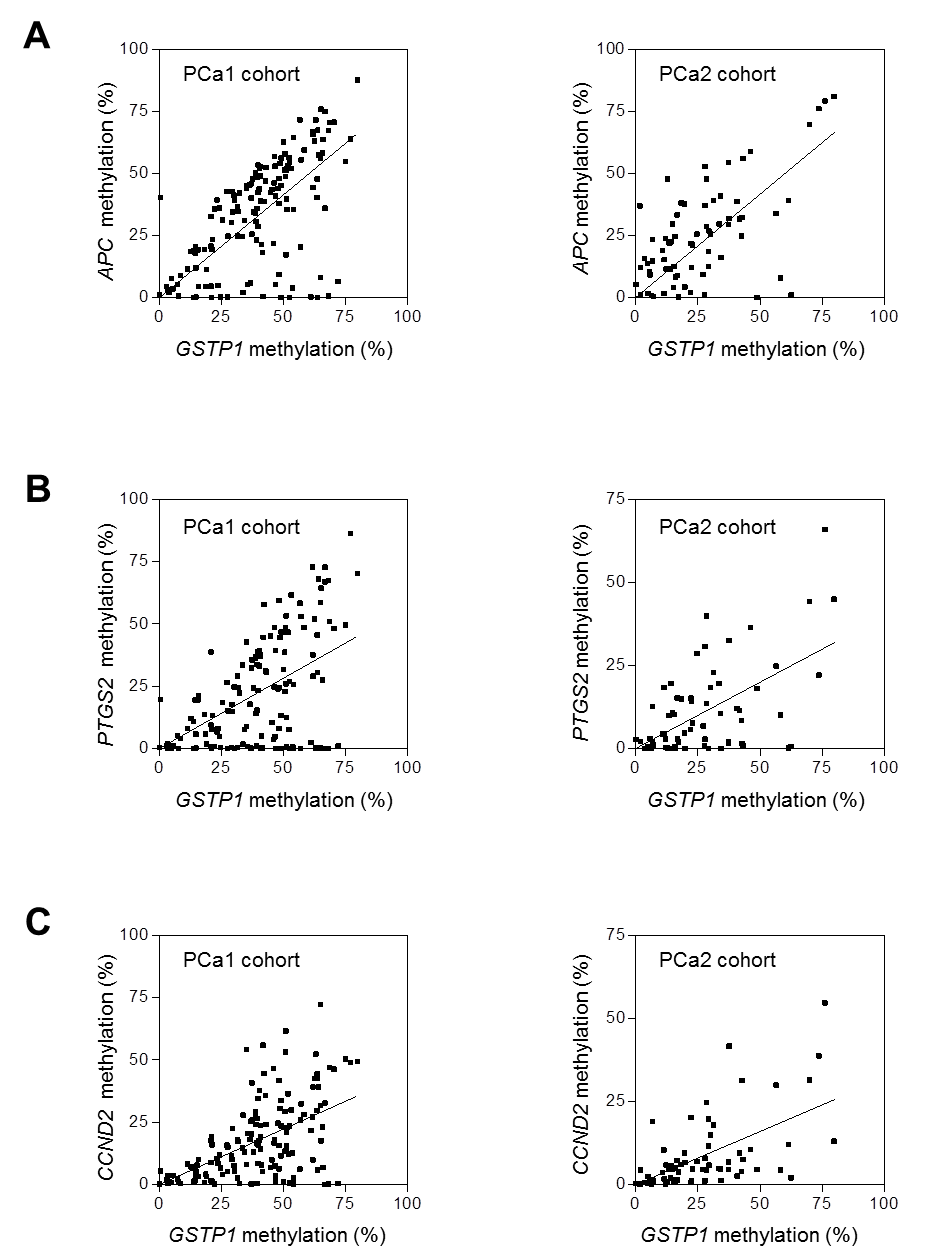
**

**Supplementary Figure C.** Scatter plots of the correlation between the methylation of GSTP1 and APC (A), PTGS2 (B) or CCND2 (C) in PCa1 (left panel) and PCa2 (right panel).

**Figure D**

70

0

35

Methylation (%)

***GSTP1***

*APC*

*RARB*

*PTGS2*

*CCND2*

***APC***

*GSTP1*

*RARB*

*PTGS2*

*CCND2*

***RARB***

*GSTP1*

*APC*

*PTGS2*

*CCND2*

***PTGS2***

*GSTP1*

*APC*

*RARB*

*CCND2*

***CCND2***

*GSTP1*

*APC*

*RARB*

*PTGS2*

**A**

**B**

**C**

**D**

**E**

**Supplementary Figure D.** Color-coded representation of DNA methylation in tumors from the combined PCa1 + PCa2 cohort (n=217). The patients were ranked according to their GSTP1 (A), APC (B), RARB (C), PTGS2 (D) or CCND2 (E) methylation values. The % of the other 4 markers is also shown. Grey boxes indicate missing values.

**Figure E**

**Clinically localized prostate cancer classified into three risk-groups based upon clinico-pathological parameters**

**Low risk**

**Intermediate risk**

**High risk**

LM

MM

HM

**Supplementary Figure E.** Schematic representation of further risk-stratification of high-risk prostate cancer patients based upon the GSTP1 methylation level. LM, GSTP11 methylation <15%; MM, GSTP1 methylation 15%-50%; HM, GSTP1 methylation >50%.

**Supplementary Tables**

|  | **Table A.** Primer pairs for the QM-MSP assay | | |  |
| --- | --- | --- | --- | --- |
|  |  |  |  |  |
|  | **Primer ID** | **Sequence 5’--3’** | **Fragment length, bp** |  |
|  | *GSTP1*-MI F | GGCGGGATTATTTTTATAAGGTT | 124 |  |
|  | *GSTP1*-MI R | CTAAAAACTCTAAACCCCATCC |  |  |
|  | *GSTP1*-MSP F | GGAGGTCGCGAGGTTTTC | 52 |  |
|  | *GSTP1*-MSP R | CTAATAACGAAAACTACGACGACGA |  |  |
|  | *GSTP1*-USP F | GTTTGGAGGTTGTGAGGTTTTT | 64 |  |
|  | *GSTP1*-USP R | CATACTCACTAATAACAAAAACTACAACAACA |  |  |
|  | *RARB*-MI F | AGGGAGAGAAGTTGGTGTTT | 172 |  |
|  | *RARB*-MI R | CCCAACAACCCTACAAAAA |  |  |
|  | *RARB*-MSP F | TAACGTGAGTTAGGAGTAGCGTTTC | 110 |  |
|  | *RARB*-MSP R | GAACGCACAAACCGACG |  |  |
|  | *RARB*-USP F | GTGTTTAATGTGAGTTAGGAGTAGTGTTTT | 121 |  |
|  | *RARB*-USP R | AACAACAAACACACAAACCAACA |  |  |
|  | *APC*-MI F | GGGAAGCGGAGAGAGAAGTAGT | 133 |  |
|  | *APC*-MI R | CGAACTACACCAATACAACCACATA |  |  |
|  | *APC*-MSP F | TATTTTCGTCGGGAGTTCGTC | 66 |  |
|  | *APC*-MSP R | CAATACAACCACATATCGATCACG |  |  |
|  | *APC*-USP F | GTTTTTTATTTTTGTTGGGAGTTTGTT | 72 |  |
|  | *APC*-USP R | CAATACAACCACATATCAATCACATACA |  |  |
|  | *CCND2*-MI F | TTTTGTAAAGATAGTTTTGATTTAAGTATG | 109 |  |
|  | *CCND2*- MI R | ACTCGCCAAACTTTCTCCCTA |  |  |
|  | *CCND2*-MSP F | CGTTAGAGTACGTGTTAGGGTCGATC | 70 |  |
|  | *CCND2*-MSP R | ACTTTCTCCCTAAAAACCGACTACG |  |  |
|  | *CCND2*-USP F | AAGTATGTGTTAGAGTATGTGTTAGGGTTGATT | 80 |  |
|  | *CCND2*-USP R | CAAACTTTCTCCCTAAAAACCAACTACA |  |  |
|  | *PTGS2*-MI F | GGCGATTAGTTTAGAATTGGTTTT | 144 |  |
|  | *PTGS2*-MI R | AAAATAATCCCCACTCTCCTATCTA |  |  |
|  | *PTGS2*-MSP F | ATTGGTTTTCGGAAGCGTTC | 81 |  |
|  | *PTGS2*-MSP R | TCCACCGCCCCAAACG |  |  |
|  | *PTGS2*-USP F | GTTTAGAATTGGTTTTTGGAAGTGTTT | 91 |  |
|  | *PTGS2*-USP R | AATTCCACCACCCCAAACA |  |  |
|  | F, forward primer; MI, methylation-independent; MSP, methylation-specific PCR; R, reverse primer; USP, unmethylated-specific PCR. | | |  |

|  | **Table B**. DNA methylation (%)of the five markers genes in all samples from the BPH cohort | | | | | |  |
| --- | --- | --- | --- | --- | --- | --- | --- |
|  |  |  |  |  |  |  | |
|  | **Sample** | **DNA methylation (%)** | | | | | |
|  |  | ***GSTP1*** | ***PTGS2*** | ***APC*** | ***CCND2*** | ***RARB*** | |
|  | 1 | 0.0006 | 0.1598 | 0.0010 | 0.4821 | 0.0003 | |
|  | 2 | 0.1822 | 0.1857 | 0.0007 | 0.3698 | 0.0003 | |
|  | 3 | 1.0873 | 0.0437 | 0.6718 | 0.4233 | 0.0003 | |
|  | 4 | 0.1243 | 0.0074 | 0.0377 | 0.5675 | 0.0003 | |
|  | 5 | 0.5779 | 0.0018 | 1.0113 | 0.2949 | 0.0003 | |
|  | 6 | 0.8584 | 0.1138 | 3.2620 | 0.0147 | 0.0003 | |
|  | 7 | 0.0001 | 0.6219 | 0.0002 | 0.0133 | - | |
|  | 8 | 0.2180 | 0.3485 | 0.7095 | 0.0315 | 0.0003 | |
|  | 9 | 0.0002 | 0.1512 | 0.0102 | 0.0859 | 0.0003 | |
|  | 10 | 0.0006 | 0.0194 | 1.1615 | 0.0473 | 0.0003 | |
|  | 11 | 0.0001 | 0.0043 | 0.0110 | -- | - | |
|  | 12 | 0.0001 | 0.3430 | 0.6142 | 0.3081 | 0.0000 | |
|  | 13 | 0.1621 | 0.1930 | 0.0006 | 0.2161 | 0.0000 | |
|  | 14 | 0.0437 | 0.0000 | 0.0000 | 0.3324 | 0.0000 | |
|  | 15 | 0.7187 | 0.0018 | 0.0008 | 0.0901 | 0.0000 | |
|  | 16 | 0.0172 | 0.0007 | 0.6203 | 0.2236 | 0.0000 | |
|  | 17 | 0.0640 | 0.1014 | 0.2248 | 0.1915 | 0.0000 | |
|  | 18 | 0.0000 | 0.2789 | 0.1097 | 0.3706 | 0.0098 | |
|  | 19 | 0.3928 | 0.2227 | 0.3082 | 0.2016 | 0.0087 | |
|  | 20 | 0.1413 | 0.5647 | 0.0010 | 0.1382 | 0.0000 | |
|  | 21 | 0.0165 | 0.0305 | 0.0003 | 0.1696 | 0.0000 | |
|  | 22 | 0.0007 | 0.1021 | 1.2815 | 0.1053 | 0.0000 | |
|  | 23 | 0.6414 | 0.0512 | 0.7584 | 0.2008 | 0.2144 | |
|  | 24 | 0.1839 | 0.2400 | 0.0051 | 0.1471 | 0.0001 | |
|  | 25 | 0.3609 | 0.0779 | 0.4031 | 0.2480 | 0.0229 | |
|  | 26 | 0.0001 | 0.3907 | 0.5317 | 0.5964 | 0.0003 | |
|  | 27 | 0.1027 | 0.0313 | 0.2809 | 0.1124 | 0.0002 | |
|  | 28 | 0.0000 | 0.0085 | 0.1641 | 0.1895 | 0.0000 | |
|  | 29 | 0.0000 | 1.3893 | 0.4974 | - | - | |
|  | 30 | 0.1846 | 0.0006 | 0.4994 | 0.0517 | 0.0000 | |
|  | 31 | 0.0837 | 0.0452 | 0.0972 | 0.2255 | 0.0000 | |
|  | 32 | 0.0078 | 0.0081 | 0.2688 | 0.1141 | 0.0000 | |
|  | 33 | 0.0000 | 1.4687 | 0.0001 | 0.0181 | 0.0010 | |
|  | 34 | 0.0001 | 0.0660 | 0.0005 | 0.0825 | 0.0013 | |
|  | 35 | 0.0001 | 0.0240 | 0.8958 | 0.0200 | 0.0009 | |
|  | 36 | 0.0002 | 0.6072 | 0.1467 | 0.1811 | 0.0014 | |
|  | 37 | 0.1819 | 0.4692 | 0.1045 | 0.0430 | 0.3731 | |
|  | 38 | 0.1472 | 0.0016 | 0.7798 | 0.1775 | 0.0004 | |
|  | 39 | 0.0852 | 0.3821 | 0.0219 | 0.2581 | 0.0774 | |
|  | 40 | 0.0001 | 0.1397 | 0.0003 | 0.4371 | 0.0000 | |
|  | 41 | 0.1800 | 0.3100 | 0.0003 | 0.1000 | 1.3073 | |
|  | 42 | 0.0000 | 0.0900 | 0.0003 | 0.3200 | 0.0000 | |
|  | (-) missing value. | | | | | | |

|  | **Table C**. DNA methylation (%) of the five markers genes in all samples from the PCa1 cohort | | | | |  |
| --- | --- | --- | --- | --- | --- | --- |
|  |  |  |  |  |  |  |
|  | **Sample** | **DNA methylation (%)** | | | | |
|  |  | ***GSTP1*** | ***PTGS2*** | ***APC*** | ***CCND2*** | ***RARB*** |
|  | 1 | 50.6558 | 38.6204 | 51.3769 | 13.1900 | 36.0167 |
|  | 2 | 63.1620 | 0.1973 | 71.4967 | 52.2560 | 83.2073 |
|  | 3 | 8.7909 | 4.0680 | 8.9330 | 0.3738 | 0.1782 |
|  | 4 | 74.9298 | 49.6151 | 54.7010 | 50.3755 | 35.0567 |
|  | 5 | 14.7570 | 6.0788 | 20.5105 | 3.2313 | 4.1488 |
|  | 6 | 53.6668 | 0.6600 | 3.9554 | 12.5847 | 41.0060 |
|  | 7 | 37.6242 | 35.5542 | 47.6347 | 25.9056 | 39.0973 |
|  | 8 | 46.5263 | 48.4602 | 56.7361 | 18.1150 | 49.3186 |
|  | 9 | 32.4961 | 22.4026 | 42.0450 | 0.4229 | 31.5814 |
|  | 10 | 40.8128 | 37.0921 | 48.9778 | 23.6717 | 34.7734 |
|  | 11 | 34.3368 | 7.5696 | 31.0203 | 13.1081 | 12.2228 |
|  | 12 | 4.1681 | 0.1417 | 1.9768 | 1.2882 | 0.9375 |
|  | 13 | 30.3997 | 0.0779 | 39.3098 | 5.4744 | 41.1274 |
|  | 14 | 63.7144 | 0.3255 | 0.0316 | 5.6789 | 28.8103 |
|  | 15 | 68.2475 | 0.0298 | 0.6697 | 0.0444 | 50.9024 |
|  | 16 | 5.2092 | 0.2015 | 7.6650 | 0.7532 | 2.4218 |
|  | 17 | 57.2060 | 52.9207 | 55.4424 | 32.3704 | 33.3627 |
|  | 18 | 29.2899 | 0.3328 | 30.2748 | 11.3559 | 22.5840 |
|  | 19 | 47.8940 | 23.9946 | 43.9172 | 24.6246 | 46.5599 |
|  | 20 | 54.2422 | 0.1459 | 35.5712 | 0.1808 | 64.1272 |
|  | 21 | 5.6200 | 1.1688 | 3.4924 | 3.5053 | 5.5179 |
|  | 22 | 35.5859 | 0.7901 | 5.2296 | 20.5150 | 37.3797 |
|  | 23 | 64.1229 | 68.0481 | 57.3887 | 39.0427 | 72.7329 |
|  | 24 | 51.3767 | 0.6884 | 39.6941 | 15.4002 | 52.2604 |
|  | 25 | 13.9267 | 10.7668 | 18.8594 | 6.3715 | 14.4148 |
|  | 26 | 14.5964 | 19.3764 | 17.9524 | 1.6451 | 10.8470 |
|  | 27 | 37.3083 | 32.1805 | 46.0754 | 20.2540 | 36.6063 |
|  | 28 | 64.9440 | 0.1934 | 8.0152 | 31.7973 | 76.8998 |
|  | 29 | 21.5937 | 13.2730 | 23.3538 | 1.1481 | 10.1252 |
|  | 30 | 50.7123 | 22.8827 | 57.9061 | 29.3258 | 53.6028 |
|  | 31 | 37.3424 | 90.5641 | 35.6899 | 25.5189 | 48.2304 |
|  | 32 | 61.9042 | 72.8781 | 66.6706 | 27.0253 | 73.9470 |
|  | 33 | 65.0137 | 58.6415 | 56.0241 | 72.2051 | 74.4128 |
|  | 34 | 35.2922 | 8.6723 | 44.1077 | 8.0678 | 12.7913 |
|  | 35 | 14.8575 | 5.7245 | 11.9894 | 3.8164 | 19.5195 |
|  | 36 | 39.4186 | 15.3479 | 48.3298 | 0.4929 | 43.3133 |
|  | 37 | 63.8842 | 30.5246 | 67.3429 | 42.7123 | 65.0628 |
|  | 38 | 31.6991 | 29.1022 | 36.6970 | 17.9804 | 35.2208 |
|  | 39 | 63.6180 | 0.1371 | 47.6774 | 44.3498 | 0.6116 |
|  | 40 | 36.5931 | 17.8030 | 45.4141 | 18.1154 | 34.5148 |
|  | 41 | 52.3163 | 7.6310 | 56.4765 | 12.2025 | 41.2008 |
|  | 42 | 33.5186 | 33.4720 | 24.6225 | 14.6811 | 42.9718 |
|  | 43 | 46.6429 | 4.6198 | 52.8156 | 7.4516 | 53.5040 |
|  | 44 | 11.5146 | 8.0192 | 11.5314 | 8.2080 | 11.0418 |
|  | 45 | 21.3630 | 7.5861 | 19.3716 | 15.9067 | 19.4564 |
|  | 46 | 52.6638 | 26.9039 | 54.2937 | 23.4395 | 33.1317 |
|  | 47 | 58.4518 | 48.5384 | 59.4805 | 28.0154 | 4.0532 |
|  | 48 | 39.1168 | 14.0349 | 30.6068 | 24.1228 | 41.4779 |
|  | 49 | 21.0684 | 9.3576 | 32.6539 | 8.8989 | 13.2028 |
|  | 50 | 51.1047 | 53.2027 | 17.2138 | 13.8237 | 20.0741 |
|  | 51 | 39.4276 | 33.7926 | 35.8835 | 26.5710 | 30.3339 |
|  | 52 | 7.5036 | 5.2599 | 5.3135 | 2.3225 | 2.4005 |
|  | 53 | 52.5902 | 0.0043 | 0.0005 | 0.6526 | 66.3483 |
|  | 54 | 15.3266 | 19.2505 | 18.9793 | 7.7453 | 14.7295 |
|  | 55 | 40.5617 | 39.1648 | 52.0657 | 37.8051 | 54.0517 |
|  | 56 | 61.9762 | 29.0815 | 65.7816 | 9.9052 | 73.1808 |
|  | 57 | 68.2716 | 67.4643 | 67.1923 | 0.4546 | 24.1433 |
|  | 58 | 42.9545 | 31.1738 | 46.9691 | 35.7020 | 26.4639 |
|  | 59 | 27.3462 | 26.5079 | 42.7169 | 15.8202 | 28.8203 |
|  | 60 | 3.0334 | 1.1307 | 4.5772 | 2.0545 | 3.5084 |
|  | 61 | 49.2280 | 46.8232 | 56.2039 | 33.6197 | 60.2080 |
|  | 62 | 15.9449 | 21.0661 | 4.3553 | 10.0734 | 18.0831 |
|  | 63 | 41.9587 | 44.6626 | 0.3890 | 55.8371 | 70.4406 |
|  | 64 | 0.6018 | 19.6772 | 40.3091 | 5.4691 | 0.0000 |
|  | 65 | 23.8350 | 7.9958 | 4.8565 | 8.4284 | 22.3199 |
|  | 66 | 51.9649 | 46.6773 | 53.1609 | 36.3346 | 52.4729 |
|  | 67 | 76.9796 | 86.2566 | 63.8904 | 48.9722 | 86.4623 |
|  | 68 | 49.2237 | 13.2056 | 45.1083 | 23.3719 | 60.1518 |
|  | 69 | 38.7841 | 36.6394 | 34.0366 | 27.3647 | 34.2744 |
|  | 70 | 30.3185 | 24.6814 | 41.1731 | 7.6654 | 23.1052 |
|  | 71 | 39.9518 | 23.2501 | 28.5107 | 15.7814 | 3.9010 |
|  | 72 | 48.5870 | 44.5098 | 53.9385 | 30.4777 | 51.6957 |
|  | 73 | 53.2336 | 61.5952 | 51.9329 | 29.9748 | 16.9239 |
|  | 74 | 38.0244 | 24.3066 | 34.5246 | 13.0008 | 29.2304 |
|  | 75 | 24.4571 | 4.8977 | 36.0873 | 11.9091 | 34.2190 |
|  | 76 | 12.9975 | 11.9353 | 18.6541 | 7.0633 | 26.8006 |
|  | 77 | 29.3453 | 17.6671 | 42.7169 | 9.9404 | 21.1098 |
|  | 78 | 56.7256 | 58.2660 | 71.5463 | 6.1736 | 11.8969 |
|  | 79 | 50.9049 | 48.4238 | 48.6876 | 53.0795 | 49.3406 |
|  | 80 | 18.2939 | 13.6583 | 19.3058 | 0.7556 | 18.4519 |
|  | 81 | 48.3170 | 8.1593 | 9.2057 | 6.2645 | 37.2827 |
|  | 82 | 62.6331 | 51.8494 | 62.9982 | 42.5440 | 71.5687 |
|  | 83 | 39.2347 | 1.0002 | 23.2693 | 16.5232 | 34.5397 |
|  | 84 | 51.2722 | 12.3150 | 51.7841 | 21.0318 | 59.1489 |
|  | 85 | 20.9618 | 38.7444 | 20.6982 | 17.6794 | 32.4816 |
|  | 86 | 41.2922 | 4.9308 | 21.4720 | 7.7093 | 11.8879 |
|  | 87 | 25.5560 | 18.3877 | 31.1813 | 4.0196 | 3.2278 |
|  | 88 | 22.2709 | 0.8416 | 35.7077 | 0.1402 | 6.2137 |
|  | 89 | 27.7824 | 15.1353 | 24.7573 | 15.0285 | 14.6073 |
|  | 90 | 54.0273 | 25.6222 | 64.4222 | 2.5445 | 67.1828 |
|  | 91 | 38.9554 | 36.0500 | 50.2076 | 29.0779 | 27.6835 |
|  | 92 | 70.3601 | 48.1451 | 70.5958 | 46.2998 | 32.5721 |
|  | 93 | 3.7157 | 0.9710 | 2.7633 | 0.3749 | 0.0001 |
|  | 94 | 68.6698 | 50.9989 | 70.3379 | 46.9907 | 73.1679 |
|  | 95 | 18.8896 | 5.8730 | 4.4421 | 4.0947 | 24.3889 |
|  | 96 | 63.6425 | 45.5802 | 40.2857 | 29.6560 | 53.5942 |
|  | 97 | 20.9350 | 0.7613 | 4.6596 | 1.4584 | 23.0783 |
|  | 98 | 19.1294 | 1.8145 | 11.3693 | 3.3219 | 0.1319 |
|  | 99 | 14.9048 | 0.1158 | 0.1842 | 5.4457 | 20.1074 |
|  | 100 | 46.2279 | 3.6896 | 21.6829 | 46.4170 | 33.4738 |
|  | 101 | 39.8861 | 38.8158 | 42.2774 | 8.3895 | 0.7174 |
|  | 102 | 79.7876 | 70.1097 | 87.5617 | 49.3757 | - |
|  | 103 | 21.2315 | 0.0012 | 0.0352 | 19.2649 | 18.2484 |
|  | 104 | 3.5485 | 1.7213 | 3.2890 | 0.5488 | 1.0980 |
|  | 105 | 29.6182 | 14.8660 | 34.3859 | 22.5004 | 26.5846 |
|  | 106 | 35.3200 | 42.8253 | 44.8778 | 54.1043 | 40.6821 |
|  | 107 | 33.8854 | 1.7097 | 1.9586 | 27.8856 | 0.1953 |
|  | 108 | 46.9831 | 4.8631 | 40.8594 | 7.6340 | 52.4426 |
|  | 109 | 37.1606 | 0.0686 | 40.1664 | 0.2330 | 52.9144 |
|  | 110 | 3.5191 | 0.0018 | 2.7674 | 3.4154 | 0.1482 |
|  | 111 | 66.8026 | 66.8805 | 74.9407 | 32.7465 | 75.3226 |
|  | 112 | 46.8792 | 39.3661 | 5.2831 | 3.5890 | 53.6186 |
|  | 113 | 43.0980 | 30.7163 | 52.4784 | 24.0793 | 45.5985 |
|  | 114 | 31.9004 | 24.4364 | 34.6210 | 17.3361 | 29.2814 |
|  | 115 | 41.7178 | 0.1690 | 18.1580 | 5.2435 | 12.4005 |
|  | 116 | 49.1311 | 0.0385 | 0.0387 | 11.5037 | 19.5479 |
|  | 117 | 40.7897 | 37.7385 | 52.7140 | 19.1222 | 42.3648 |
|  | 118 | 46.3979 | 24.6892 | 46.1827 | 16.6203 | 38.7641 |
|  | 119 | 72.0019 | 1.0012 | 6.5388 | 0.2790 | 75.6104 |
|  | 120 | 48.3847 | 24.4649 | 37.8094 | 15.7349 | 34.1574 |
|  | 121 | 14.0885 | 0.0053 | 0.0009 | 1.3706 | 0.0000 |
|  | 122 | 65.9684 | 0.0498 | 63.7067 | 6.8828 | 67.3333 |
|  | 123 | 23.3525 | 6.7259 | 39.2976 | 10.4093 | 21.0953 |
|  | 124 | 48.3044 | 59.4475 | 48.0045 | 41.6064 | 55.4290 |
|  | 125 | 27.5728 | 0.0218 | 0.2327 | 5.2037 | 0.0501 |
|  | 126 | 15.9800 | 19.8705 | 19.3329 | 3.1935 | 23.2870 |
|  | 127 | 46.9557 | 0.0009 | 52.9009 | 1.4076 | 11.4169 |
|  | 128 | 0.1948 | 0.3029 | 0.9671 | 0.2389 | 0.0000 |
|  | 129 | 61.9589 | 37.5723 | 44.3310 | 39.1241 | 65.9202 |
|  | 130 | 44.7551 | 45.0827 | 42.3405 | 6.8066 | 45.4555 |
|  | 131 | 61.1616 | 2.3727 | 0.3075 | 21.6169 | 0.8008 |
|  | 132 | 56.9429 | 0.0003 | 20.3109 | 25.9058 | 0.0631 |
|  | 133 | 7.8913 | 0.0460 | 0.7303 | 1.2968 | 0.0000 |
|  | 134 | 65.1862 | 64.3376 | 75.8457 | 17.5161 | 62.9566 |
|  | 135 | 50.0894 | 31.8164 | 62.5163 | 1.0687 | 52.7124 |
|  | 136 | 40.3423 | 33.0071 | 43.5931 | 17.7104 | 29.0000 |
|  | 137 | 40.8398 | 0.2197 | 38.9629 | 14.2616 | 9.6448 |
|  | 138 | 45.4244 | 10.3600 | 43.4454 | 9.8372 | 32.0618 |
|  | 139 | 31.5554 | 0.3180 | 24.8236 | 2.6013 | 31.7289 |
|  | 140 | 42.3457 | 57.6748 | 38.6532 | 44.5191 | 59.2821 |
|  | 141 | 51.0603 | 25.9398 | 51.2452 | 61.6216 | 60.6978 |
|  | 142 | 36.9181 | 0.3097 | 5.8671 | 25.3952 | 42.4370 |
|  | 143 | 24.8655 | 0.4650 | 0.3047 | 6.6370 | 17.7036 |
|  | 144 | 65.8045 | 27.3591 | 58.1955 | 23.0256 | 43.5988 |
|  | 145 | 51.4265 | 46.4173 | 35.5293 | 22.7449 | 60.4297 |
|  | 146 | 39.8619 | 37.4180 | 53.4120 | 34.4595 | 28.9733 |
|  | 147 | 66.7897 | 72.7824 | 36.0455 | 0.0322 | 28.9208 |
|  | (-) missing value. | | | | | |

|  | **Table D.** DNA methylation (%) of the five markers genes in all samples from the PCa2 cohort | | | | |  |
| --- | --- | --- | --- | --- | --- | --- |
|  |  |  | | | | |
|  | **Sample** | **DNA methylation (%)** | | | | |
|  |  | ***GSTP1*** | ***PTGS2*** | ***APC*** | ***CCND2*** | ***RARB*** |
|  | 1 | 16.2063 | 0.0025 | 8.2831 | 4.9334 | 0.0027 |
|  | 2 | 34.3189 | 10.4399 | 40.8147 | 4.7323 | 33.7850 |
|  | 3 | 12.2265 | 2.8180 | 23.7146 | 0.1269 | 18.0101 |
|  | 4 | 7.0600 | 12.6109 | 23.4090 | 18.8641 | 12.8895 |
|  | 5 | 29.6610 | - | 26.8238 | 5.7842 | 22.1939 |
|  | 6 | 6.6347 | 0.0000 | 0.7450 | 0.0873 | 0.0000 |
|  | 7 | 1.9753 | - | 36.8609 | 0.0000 | - |
|  | 8 | 37.4927 | 2.0467 | 29.3757 | 4.5608 | 31.5217 |
|  | 9 | 69.8520 | 44.2023 | 69.6975 | 31.4057 | 68.8133 |
|  | 10 | 11.5789 | 18.3736 | 1.6761 | 10.3722 | 0.0089 |
|  | 11 | 31.5087 | 22.9196 | 39.1061 | 17.8526 | 21.8874 |
|  | 12 | 43.3990 | 0.7941 | 55.9318 | 7.5824 | 48.0767 |
|  | 13 | 2.2166 | 2.1230 | 12.0373 | 0.1564 | 8.4833 |
|  | 14 | 29.4697 | - | 18.5795 | 19.7853 | - |
|  | 15 | 46.4101 | 36.4033 | 58.6945 | 10.5623 | 45.4769 |
|  | 16 | - | - | - | - | - |
|  | 17 | 11.7194 | 4.4310 | 15.1895 | 1.6501 | 8.3382 |
|  | 18 | 12.9703 | 0.0644 | 47.5999 | 0.9641 | 23.7472 |
|  | 19 | 19.7672 | 4.7385 | 37.6287 | 9.4887 | 35.8606 |
|  | 20 | 28.0245 | 2.8521 | 36.9515 | 1.2023 | 13.4929 |
|  | 21 | 17.3447 | 1.8976 | 0.2370 | 3.7270 | 2.4198 |
|  | 22 | 22.2551 | 5.6644 | 1.9215 | 0.5769 | 18.4222 |
|  | 23 | 76.1108 | 65.8910 | 79.1471 | 54.6355 | 77.8201 |
|  | 24 | 16.8708 | 15.2357 | 33.3118 | 7.2327 | 16.9569 |
|  | 25 | 40.9435 | 12.1222 | 38.6050 | 2.5454 | 37.1358 |
|  | 26 | 13.6877 | 9.8827 | 21.8935 | 4.7271 | 6.7106 |
|  | 27 | 16.9831 | 2.9259 | 8.9992 | 1.3502 | 3.5605 |
|  | 28 | 18.5273 | 14.8992 | 38.0274 | 6.0855 | 20.1300 |
|  | 29 | 27.7637 | 0.7045 | 0.9866 | 1.0791 | 32.3300 |
|  | 30 | 14.3223 | 0.0000 | 11.5607 | 1.9295 | 0.0000 |
|  | 31 | 5.2509 | 0.5421 | 1.5855 | 2.3763 | 0.0196 |
|  | 32 | 6.0303 | 2.9780 | 9.1295 | 1.4813 | 3.9602 |
|  | 33 | 22.3637 | 15.1691 | 21.7176 | 20.2113 | 23.9848 |
|  | 34 | 33.7362 | 19.5105 | 29.6236 | 4.8431 | 31.2912 |
|  | 35 | 0.3452 | 2.6333 | 5.2996 | 0.2484 | 7.1159 |
|  | 36 | 62.5600 | 0.6174 | 1.0421 | 2.0563 | 57.3774 |
|  | 37 | 34.5780 | 0.0003 | 16.1910 | 1.1617 | 0.0030 |
|  | 38 | 37.5802 | 1.5255 | 54.2860 | 6.6941 | 25.2346 |
|  | 39 | 24.8300 | 28.6019 | 25.5476 | 6.8800 | 28.0050 |
|  | 40 | 14.9406 | 10.7702 | 29.6216 | 4.4902 | 6.6641 |
|  | 41 | 27.2945 | 6.7534 | 9.2313 | 4.5616 | 23.3282 |
|  | 42 | 28.7473 | 39.7800 | 47.6497 | 24.6487 | 30.1340 |
|  | 43 | 15.3415 | 0.0002 | 3.9586 | 0.7355 | 0.0000 |
|  | 44 | 4.9630 | 0.0000 | 13.8282 | 0.3556 | 0.0153 |
|  | 45 | 3.8325 | 0.0054 | 15.6231 | 0.6809 | 2.0802 |
|  | 46 | 28.7001 | 13.4828 | 28.5514 | 4.3727 | 18.1170 |
|  | 47 | 16.1223 | 0.6049 | 24.5027 | 1.1054 | 17.7680 |
|  | 48 | 11.0389 | 4.3057 | 18.8307 | 3.5757 | 11.7577 |
|  | 49 | 15.5694 | 10.2751 | 12.4998 | 1.3644 | 14.3477 |
|  | 50 | 41.9693 | 11.4512 | 31.6146 | 9.4917 | 36.3460 |
|  | 51 | 48.8117 | 18.0536 | 0.0356 | 4.4741 | 5.4579 |
|  | 52 | 28.0114 | 30.6313 | 52.7188 | 7.9074 | 42.7811 |
|  | 53 | 61.5258 | 0.1127 | 38.9200 | 12.1009 | 42.3700 |
|  | 54 | 56.5631 | 24.7598 | 33.7934 | 29.9301 | 45.9169 |
|  | 55 | 30.2100 | 18.3190 | 25.5872 | 14.8798 | 39.8315 |
|  | 56 | 14.4601 | 19.5096 | 22.4870 | 5.5857 | 20.3644 |
|  | 57 | 7.4866 | 1.0064 | 0.2692 | 1.0661 | 0.0063 |
|  | 58 | 19.9553 | 0.0231 | 4.2672 | 6.6615 | 0.0092 |
|  | 59 | 29.5635 | 0.0000 | 12.4200 | 11.6328 | - |
|  | 60 | 22.6179 | 14.0754 | 12.0155 | 1.0844 | - |
|  | 61 | 12.4550 | 0.2626 | 11.4880 | 5.8135 | 10.3082 |
|  | 62 | 42.8750 | 1.4975 | 32.0953 | 31.3605 | 53.0244 |
|  | 63 | 42.6070 | 8.3658 | 24.6787 | 3.9600 | 24.2655 |
|  | 64 | 5.7989 | 0.0219 | 10.3755 | 0.5019 | 0.0000 |
|  | 65 | 58.3274 | 10.0206 | 7.8122 | 4.3625 | 26.4868 |
|  | 66 | 6.9602 | 2.0347 | 14.6164 | 1.4075 | 11.8887 |
|  | 67 | 23.1376 | 7.6672 | 21.2471 | 4.6436 | 20.6233 |
|  | 68 | 2.2523 | - | 1.2546 | 4.2577 | 0.0000 |
|  | 69 | 73.7291 | 22.1026 | 75.9757 | 38.7255 | 69.7786 |
|  | 70 | 79.7878 | 44.9146 | 80.8234 | 13.0185 | 67.5041 |
|  | 71 | 37.7365 | 32.5500 | 31.8154 | 41.6467 | 29.4000 |
|  | (-) missing value. | | | | | |

|  | **Table E.** Diagnostic information of DNA methylation for the five-gene panel | | | | | | | | | | | | | | | |  |
| --- | --- | --- | --- | --- | --- | --- | --- | --- | --- | --- | --- | --- | --- | --- | --- | --- | --- |
|  |  |  |  |  |  | |  | |  | | |  | | |  | |  |
|  |  |  | **Specificity** |  | **Sensitivity** | |  | | **PPV** | | |  | | | **NPV** | |  |
|  | **Gene (combination) / Cohort** |  | **BPH** |  | **PCa1** | **PCa2** | |  | | **PCa1** | **PCa2** | |  | **PCa1** | | **PCa2** |  |
|  | *RARB* |  | 1.00 |  | 0.89 | 0.82 | |  | | 1.00 | 1.00 | |  | 0.71 | | 0.76 |  |
|  | *GSTP1* |  | 1.00 |  | 0.99 | 0.97 | |  | | 1.00 | 1.00 | |  | 0.96 | | 0.95 |  |
|  | *APC* |  | 0.98 |  | 0.90 | 0.86 | |  | | 0.99 | 0.98 | |  | 0.73 | | 0.80 |  |
|  | *CCND2* |  | 1.00 |  | 0.89 | 0.84 | |  | | 1.00 | 1.00 | |  | 0.71 | | 0.78 |  |
|  | *PTGS2* |  | 1.00 |  | 0.72 | 0.67 | |  | | 1.00 | 1.00 | |  | 0.51 | | 0.66 |  |
|  | *GSTP1/APC* |  | 0.98 |  | 0.99 | 1.00 | |  | | 0.99 | 0.98 | |  | 0.98 | | 1.00 |  |
|  | *GSTP1/APC/CCND2/RARB/PTGS2* |  | 0.98 |  | 0.99 | 1.00 | |  | | 0.99 | 0.98 | |  | 0.98 | | 1.00 |  |
|  | The methylation cutoff was set at 2% for all genes, except for *CCND2* (1%). BPH, 42 patients with benign prostatic hyperplasia, NPV, negative predictive value; PPV, positive predictive value; Training cohort, 71 patients with high-risk prostate cancer; Validation cohort, 147 patients with high-risk prostate cancer. | | | | | | | | | | | | | | | |  |

|  | **Table F**. Scoring of the immunostainings for ERG and GSTP1 via All method and stroma content (%) in PCa2 cohort | | | |
| --- | --- | --- | --- | --- |
|  |  |  |  |  |
|  | **Sample** | **ERG*** | **GSTP1*** | **Stroma (%)** |
|  | 1 | 4 (1+3) | 0 | 20 |
|  | 2 | 6 (1+5) | 0 | 15 |
|  | 3 | 0 | 0 | 20 |
|  | 4 | 0 | 0 | 20 |
|  | 5 | 6 (2+4) | 6 (3+3) | 35 |
|  | 6 | 0 | 0 | 30 |
|  | 7 | 0 | 0 | 10 |
|  | 8 | 3 (1+2) | 0 | 60 |
|  | 9 | 6 (2+4) | 0 | 20 |
|  | 10 | 4 (1+3) | 0 | 35 |
|  | 11 | 4 (1+3) | 0 | 30 |
|  | 12 | - | - | - |
|  | 13 | 5 (1+4) | 0 | 25 |
|  | 14 | 6 (2+4) | 0 | 20 |
|  | 15 | 0 | 0 | 15 |
|  | 16 | 7 (2+5) | 0 | 15 |
|  | 17 | 5 (1+4) | 0 | 15 |
|  | 18 | 0 | 0 | 20 |
|  | 19 | 0 | 0 | 20 |
|  | 20 | 8 (3+5) | 0 | 30 |
|  | 21 | 5 (1+4) | 0 | 25 |
|  | 22 | 0 | 0 | 20 |
|  | 23 | 0 | 0 | 10 |
|  | 24 | 7 (2+5) | 0 | 10 |
|  | 25 | 7 (2+5) | 0 | 20 |
|  | 26 | 7 (2+5) | 0 | 30 |
|  | 27 | 7 (2+5) | 0 | 20 |
|  | 28 | 0 | 0 | 20 |
|  | 29 | 8 (3+5) | 0 | 10 |
|  | 30 | 5 (1+4) | 0 | 30 |
|  | 31 | 0 | 0 | 20 |
|  | 32 | 6 (2+4) | 0 | 25 |
|  | 33 | 0 | 0 | 30 |
|  | 34 | 6 (2+4) | 0 | 30 |
|  | 35 | 0 | 0 | 30 |
|  | 36 | 8 (3+5) | 0 | 20 |
|  | 37 | 7 (2+5) | 0 | 30 |
|  | 38 | 0 | 0 | 25 |
|  | 39 | 0 | 0 | 25 |
|  | 40 | 0 | 0 | 15 |
|  | 41 | 8 (3+5) | 0 | 20 |
|  | 42 | 7 (2+5) | 0 | 30 |
|  | 43 | 0 | 0 | 15 |
|  | 44 | 0 | 5 (2+3) | 10 |
|  | 45 | 7 (2+5) | 0 | 25 |
|  | 46 | 7 (2+5) | 0 | 20 |
|  | 47 | 7 (2+5) | 0 | 25 |
|  | 48 | 7 (2+5) | 0 | 30 |
|  | 49 | 6 (1+5) | 0 | 35 |
|  | 50 | 7 (2+5) | 4 (1+3) | 40 |
|  | 51 | 6 (1+5) | 0 | 20 |
|  | 52 | - | - | - |
|  | 53 | 7 (2+5) | 0 | 15 |
|  | 54 | 6 (2+4) | 4 (1+3) | 25 |
|  | 55 | 0 | 0 | 20 |
|  | 56 | 7 (2+5) | 0 | 20 |
|  | 57 | 0 | 0 | 25 |
|  | 58 | 0 | 0 | 20 |
|  | 59 | 4 (1+3) | 0 | 40 |
|  | 60 | 0 | 0 | 20 |
|  | 61 | 6 (2+4) | 0 | 25 |
|  | 62 | 8 (3+5) | 0 | 20 |
|  | 63 | 7 (2+5) | 0 | 15 |
|  | 64 | 8 (3+5) | 0 | 30 |
|  | 65 | 0 | 0 | 10 |
|  | 66 | 6 (1+5) | 0 | 30 |
|  | 67 | 6 (3+3) | 0 | 20 |
|  | 68 | 0 | 7 (3+4) | 30 |
|  | 69 | 0 | 0 | 15 |
|  | 70 | 0 | 0 | 20 |
|  | 71 | 5 (1+4) | 0 | 30 |
|  | * Total score = intensity score + proportion score. The intensity score was graded on a scale of 0-3: 0, no staining; 1, weak staining; 2, moderate staining ; 3, strong staining. The proportion score estimates the proportion of positive tumor cells on a scale of 0-5: 0, none; 1, 1%; 2, 1-10%; 3, 10-33%; 4, 33-66%; 5, 66-100%. | | | |

|  |  | **Table G. Correlation analysis between ERG staining, GSTP1 staining, stroma content and GSTP1 DNA methylation** | | | | | | | | | | |
| --- | --- | --- | --- | --- | --- | --- | --- | --- | --- | --- | --- | --- |
|  |  | **Variable** | | ***n*** |  | ***GSTP1 methylation*** | | | | | | |
|  |  |  | |  |  | **Continuous (%)** |  | **Trichotomized (*n*)** | | | | |
|  |  |  | |  |  | **M ± SD** |  | **LM** | | **MM** | | **HM** |
|  |  | ERG negative* | | 26 |  | 25 ± 24 |  | 11 | | 11 | | 4 |
|  |  | ERG positive * | | 42 |  | 27 ± 17 |  | 12 | | 26 | | 4 |
|  |  | *P*-value | |  |  | 0.219** |  | 0.257^$^ | | | | |
|  |  |  |  |  |  |  |  |  |  | |  | |
|  |  | GSTP1 negative* | | 63 |  | 26 ± 19 |  | 21 | | 35 | | 7 |
|  |  | GSTP1 positive* | | 5 |  | 27 ± 24 |  | 2 | | 2 | | 1 |
|  |  | *P*-value | |  |  | 1.000** |  | 0.529^$^ | | | | |
|  |  |  | |  |  |  |  |  | | | | |
|  |  | Stroma <15% | | 6 |  | 31± 30 |  | 2 | | 2 | | 2 |
|  |  | Stroma *≥*15% | | 62 |  | 26 ± 18 |  | 21 | | 35 | | 6 |
|  |  | *P*-value | |  |  | 0.974** |  | 0.190^$^ | | | | |
|  |  | *, The proportion and intensity scores of the Allred score were summed to obtain the total scores of 0, 2-8. A score of 0-2 was considered as negative and 3-8 as positive. **, Mann-Whitney U-test; ^$^, Fisher exact test; *n*, number; M, mean; SD, Standard deviation; LM, low methylation; MM, moderate methylation; HM, high methylation. | | | | | | | | | | |

|  | **Table H.** Pearson correlations coefficients of promoter methylation of the five-gene panel (all significant at *P* <0.001) | | | | | | |  |
| --- | --- | --- | --- | --- | --- | --- | --- | --- |
|  |  |  |  | | | | |  |
|  |  |  | **Gene** | | | | |  |
|  | **Gene** | **Cohort** | ***RARB*** | ***GSTP1*** | ***APC*** | ***CCND2*** | ***PTGS2*** |  |
|  | *RARB* | PCa1 | 1 | 0.68 | 0.52 | 0.50 | 0.45 |  |
|  |  | PCa2 | 1 | 0.82 | 0.73 | 0.67 | 0.60 |  |
|  | *GSTP1* | PCa1 | 0.68 | 1 | 0.62 | 0.53 | 0.52 |  |
|  |  | PCa2 | 0.82 | 1 | 0.51 | 0.59 | 0.51 |  |
|  | *APC* | PCa1 | 0.52 | 0.62 | 1 | 0.48 | 0.65 |  |
|  |  | PCa2 | 0.73 | 0.51 | 1 | 0.58 | 0.63 |  |
|  | *CCND2* | PCa1 | 0.50 | 0.53 | 0.48 | 1 | 0.65 |  |
|  |  | PCa2 | 0.67 | 0.59 | 0.58 | 1 | 0.73 |  |
|  | *PTGS2* | PCa1 | 0.45 | 0.48 | 0.65 | 0.65 | 1 |  |
|  |  | PCa2 | 0.60 | 0.58 | 0.63 | 0.73 | 1 |  |
|  | PCa 1, training cohort, 147 patients with high-risk prostate cancer; PCa2; validation cohort, 71 patients with high-risk prostate cancer. | | | | | | |  |

|  | **Table I.** Pairwise comparisons of DNA methylation and Gleason scores in PCa1 | | | | | |  |
| --- | --- | --- | --- | --- | --- | --- | --- |
|  |  |  |  | |  |  |  |
|  | **Cohort** | **Variable** | **Gleason score** | |  | ***P*-values** |  |
|  |  |  | **Group 1** | **Group 2** |  |  | |
|  | **PCa1** | ***GSTP1*** | _ | _ |  | **0.604** | |
|  |  |  | 2-6 | 7 |  | 0.303 | |
|  |  |  |  | 8-10 |  | 0.679 | |
|  |  |  | 7 | 8-10 |  | 0.608 | |
|  |  | ***PTGS2*** | _ | _ |  | **0.007** | |
|  |  |  | 2-6 | 7 |  | 0.040 | |
|  |  |  |  | 8-10 |  | 0.002 | |
|  |  |  | 7 | 8-10 |  | 0.459 | |
|  |  | ***APC*** | _ | _ |  | **0.135** | |
|  |  |  | 2-6 | 7 |  | 0.124 | |
|  |  |  |  | 8-10 |  | 0.067 | |
|  |  |  | 7 | 8-10 |  | 0.955 | |
|  |  | ***CCND2*** | _ | _ |  | **0.402** | |
|  |  |  | 2-6 | 7 |  | 0.345 | |
|  |  |  |  | 8-10 |  | 0.207 | |
|  |  |  | 7 | 8-10 |  | 0.725 | |
|  |  | ***RARB*** | _ | _ |  | **0.165** | |
|  |  |  | 2-6 | 7 |  | 0.158 | |
|  |  |  |  | 8-10 |  | 0.084 | |
|  |  |  | 7 | 8-10 |  | 0.688 | |
|  |  |  |  |  |  |  | |
|  | **PCa2** | ***GSTP1*** | _ | _ |  | **0.335** | |
|  |  |  | 2-6 | 7 |  | 0.296 | |
|  |  |  |  | 8-10 |  | 0.660 | |
|  |  |  | 7 | 8-10 |  | 0.189 | |
|  |  | ***PTGS2*** | _ | _ |  | **0.145** | |
|  |  |  | 2-6 | 7 |  | 0.091 | |
|  |  |  |  | 8-10 |  | 0.557 | |
|  |  |  | 7 | 8-10 |  | 0.152 | |
|  |  | ***APC*** | _ | _ |  | **0.134** | |
|  |  |  | 2-6 | 7 |  | 0.046 | |
|  |  |  |  | 8-10 |  | 0.229 | |
|  |  |  | 7 | 8-10 |  | 0.637 | |
|  |  | ***CCND2*** | _ | _ |  | **0.060** | |
|  |  |  | 2-6 | 7 |  | 0.060 | |
|  |  |  |  | 8-10 |  | 0.395 | |
|  |  |  | 7 | 8-10 |  | 0.055 | |
|  |  | ***RARB*** | _ | _ |  | **0.082** | |
|  |  |  | 2-6 | 7 |  | 0.030 | |
|  |  |  |  | 8-10 |  | 0.208 | |
|  |  |  | 7 | 8-10 |  | 0.400 | |
|  | *P*-values in bold refer to the result of the Kruskal-Wallis test. Mann-whitney U tests were used for pairwise comparisons. | | | | | | |

|  | **Table J.** Univariate and multivariate Cox regression analysis of clinical failure | | | | | | | | |  | |  |  |
| --- | --- | --- | --- | --- | --- | --- | --- | --- | --- | --- | --- | --- | --- |
|  |  |  | | | |  |  | | | |  | |  |
|  |  | **Univariate** | | | |  | **Multivariate** | | | |  | |  |
|  | **Variable** | | **HR** | **95% CI** | ***P*-value** |  | **HR** | **95% CI** | ***P*-value** | |  | |  |
|  | Cohort PCa1 |  | |  |  |  |  |  |  | |  | |  |
|  | ***GSTP1* dichotomized c=50%** | 1.68 | | 0.80-3.52 | 0.173 |  | - | - | - | |  | |  |
|  | ***GSTP1* dichotomized c=15%** | 0.39 | | 0.17-0.92 | 0.031 |  | 0.22 | 0.09-0.54 | 0.001 | |  | |  |
|  | Pathological T stage 2-3a vs 3b - 4 | 2.28 | | 0.93-5.61 | 0.072 |  | 1.77 | 0.71-4.44 | 0.224 | |  | |  |
|  | Gleason score 2-7 vs 8-10 | 3.40 | | 1.63-7.09 | 0.001 |  | 5.00 | 2.26-11.06 | <0.001 | |  | |  |
|  | Preoperative PSA continuous | 1.00 | | 1.00-1.01 | 0.640 |  | 1.00 | 0.99-1.01 | 0.676 | |  | |  |
|  | Cohort PCa2 |  | |  |  |  |  |  |  | |  | |  |
|  | ***GSTP1* dichotomized c=50%** | 3.64 | | 1.12-11.88 | 0.032 |  | 3.84 | 1.10-13.44 | 0.035 | |  | |  |
|  | ***GSTP1* dichotomized c=15%** | 0.78 | | 0.25-2.38 | 0.659 |  | - | - | - | |  | |  |
|  | Pathological T stage 2-3a vs 3b - 4 | 7.03 | | 2.14-23.09 | 0.001 |  | 6.31 | 1.85-21.48 | 0.003 | |  | |  |
|  | Gleason score 2-7 vs 8-10 | 7.49 | | 2.28-24.68 | < 0.001 |  | 8.54 | 2.30-31.77 | 0.001 | |  | |  |
|  | Preoperative PSA continuous | 1.01 | | 1.00-1.03 | 0.107 |  | 1.01 | 0.99-1.03 | 0.201 | |  | |  |
|  | Cohort PCa1 |  | |  |  |  |  |  |  | |  | |  |
|  | ***PTGS2* dichotomized c=4%** | 0.39 | | 0.18-0.81 | 0.013 |  | 0.21 | 0.09-0.50 | <0.001 | |  | |  |
|  | Pathological T stage 2-3a vs 3b - 4 | 2.28 | | 0.93-5.61 | 0.072 |  | 2.65 | 0.98-7.18 | 0.055 | |  | |  |
|  | Gleason score 2-7 vs 8-10 | 3.40 | | 1.63-7.09 | 0.001 |  | 6.56 | 2.83-15.20 | <0.001 | |  | |  |
|  | Preoperative PSA continuous | 1.00 | | 1.00-1.01 | 0.640 |  | 1.00 | 0.99-1.01 | 0.787 | |  | |  |
|  | Cohort PCa2 |  | |  |  |  |  |  |  | |  | |  |
|  | ***PTGS* dichotomized c=4%** | 1.31 | | 0.39-4.35 | 0.661 |  | - | - | - | |  | |  |
|  | Pathological T stage 2-3a vs 3b - 4 | 7.03 | | 2.14-23.09 | 0.001 |  | - | - | - | |  | |  |
|  | Gleason score 2-7 vs 8-10 | 7.49 | | 2.28-24.68 | < 0.001 |  | - | - | - | |  | |  |
|  | Preoperative PSA continuous | 1.01 | | 1.00-1.03 | 0.107 |  | - | - | - | |  | |  |
|  | Cohort PCa1 |  | |  |  |  |  |  |  | |  | |  |
|  | ***CCND2* dichotomized c=1%** | 0.44 | | 0.17-1.16 | 0.096 |  | - | - | - | |  | |  |
|  | Pathological T stage 2-3a vs 3b - 4 | 2.28 | | 0.93-5.61 | 0.072 |  | - | - | - | |  | |  |
|  | Gleason score 2-7 vs 8-10 | 3.40 | | 1.63-7.09 | 0.001 |  | - | - | - | |  | |  |
|  | Preoperative PSA continuous | 1.00 | | 1.00-1.01 | 0.640 |  | - | - | - | |  | |  |
|  | Cohort PCa2 |  | |  |  |  |  |  |  | |  | |  |
|  | ***CCND2* dichotomized c=1%** | 0.21 | | 0.07-0.65 | 0.007 |  | 0.19 | 0.05-0.79 | 0.022 | |  | |  |
|  | Pathological T stage 2-3a vs 3b - 4 | 7.03 | | 2.14-23.09 | 0.001 |  | 11.40 | 2.71-48.00 | <0.001 | |  | |  |
|  | Gleason score 2-7 vs 8-10 | 7.49 | | 2.28-24.68 | < 0.001 |  | 5.08 | 1.37-18.85 | 0.015 | |  | |  |
|  | Preoperative PSA continuous | 1.01 | | 1.00-1.03 | 0.107 |  | 1.00 | 0.99-1.02 | 0.688 | |  | |  |
|  | Cohort PCa1 |  | |  |  |  |  |  |  | |  | |  |
|  | ***RARB* dichotomized c=40%** | 0.98 | | 0.47-2.07 | 0.960 |  | - | - | - | |  | |  |
|  | Pathological T stage 2-3a vs 3b - 4 | 2.28 | | 0.93-5.61 | 0.072 |  | - | - | - | |  | |  |
|  | Gleason score 2-7 vs 8-10 | 3.40 | | 1.63-7.09 | 0.001 |  | - | - | - | |  | |  |
|  | Preoperative PSA continuous | 1.00 | | 1.00-1.01 | 0.640 |  | - | - | - | |  | |  |
|  | Cohort PCa2 |  | |  |  |  |  |  |  | |  | |  |
|  | ***RARB* dichotomized c=40%** | 3.45 | | 1.09-10.87 | 0.035 |  | 3.81 | 1.09-13.34 | 0.036 | |  | |  |
|  | Pathological T stage 2-3a vs 3b - 4 | 7.03 | | 2.14-23.09 | 0.001 |  | 4.61 | 1.29-16.48 | 0.018 | |  | |  |
|  | Gleason score 2-7 vs 8-10 | 7.49 | | 2.28-24.68 | < 0.001 |  | 9.81 | 2.39-40.33 | 0.001 | |  | |  |
|  | Preoperative PSA continuous | 1.01 | | 1.00-1.03 | 0.107 |  | 1.01 | 0.99-1.03 | 0.207 | |  | |  |
|  | Hazard Ratio (HR) >1 (<1) indicates higher (lower) risk for the second group. (%), % *GSTP1* methylation; CF, clinical failure; CI, confidence interval; HM, high methylation; LM, low methylation; MM, moderate methylation; c, cutoff. | | | | | | | | | | |  | |
